# Rapid and Flexible RT-qPCR Surveillance Platforms To Detect SARS-CoV-2 Mutations

**DOI:** 10.1128/spectrum.03591-22

**Published:** 2023-01-09

**Authors:** Katja Spiess, Vithiagaran Gunalan, Ellinor Marving, Sofie Holdflod Nielsen, Michelle G. P. Jørgensen, Anna S. Fomsgaard, Line Nielsen, Alonzo Alfaro-Núñez, Søren M. Karst, The Danish COVID-19 Genome Consortium (DCGC), Shila Mortensen, Morten Rasmussen, Ria Lassaunière, Maiken Worsøe Rosenstierne, Charlotta Polacek, Jannik Fonager, Arieh S. Cohen, Claus Nielsen, Anders Fomsgaard

**Affiliations:** a Department of Virus and Microbiological Special Diagnostics, Statens Serum Institut, Copenhagen, Denmark; b Test Center Denmark, Statens Serum Institut, Copenhagen, Denmark; c Qlife ApS Symbion, Copenhagen, Denmark; American Type Culture Collection; Universidad de Las Américas

**Keywords:** SARS-CoV-2, signature mutations, variant of concern, variant PCR, large-scale screening, whole-genome sequencing, national surveillance program, RT-qPCR platforms, variants of concern, national surveillance system

## Abstract

Multiple mutations in severe acute respiratory syndrome coronavirus 2 (SARS-CoV-2) variants of concern (VOCs) increase transmission, disease severity, and immune evasion and facilitate zoonotic or anthropozoonotic infections. Four such mutations, ΔH69/V70, L452R, E484K, and N501Y, occurred in the SARS-CoV-2 spike glycoprotein in combinations that allow the simultaneous detection of VOCs. Here, we present two flexible reverse transcription-quantitative PCR (RT-qPCR) platforms for small- and large-scale screening (also known as variant PCR) to detect these mutations and schemes for adapting the platforms to future mutations. The large-scale RT-qPCR platform was validated by pairwise matching of RT-qPCR results with whole-genome sequencing (WGS) consensus genomes, showing high specificity and sensitivity. Both platforms are valuable examples of complementing WGS to support the rapid detection of VOCs. Our mutational signature approach served as an important intervention measure for the Danish public health system to detect and delay the emergence of new VOCs.

**IMPORTANCE** Denmark weathered the SARS-CoV-2 crisis with relatively low rates of infection and death. Intensive testing strategies with the aim of detecting SARS-CoV-2 in symptomatic and nonsymptomatic individuals were available by establishing a national test system called TestCenter Denmark. This testing regime included the detection of SARS-CoV-2 signature mutations, with referral to the national health system, thereby delaying outbreaks of variants of concern. Our study describes the design of the large-scale RT-qPCR platform established at TestCenter Denmark in conjunction with whole-genome sequencing to report mutations of concern to the national health system. Validation of the large-scale RT-qPCR platform using paired WGS consensus genomes showed high sensitivity and specificity. For smaller laboratories with limited infrastructure, we developed a flexible small-scale RT-qPCR platform to detect three signature mutations in a single run. The RT-qPCR platforms are important tools to support the control of the SARS-CoV-2 endemic in Denmark.

## INTRODUCTION

**FIG 1:**
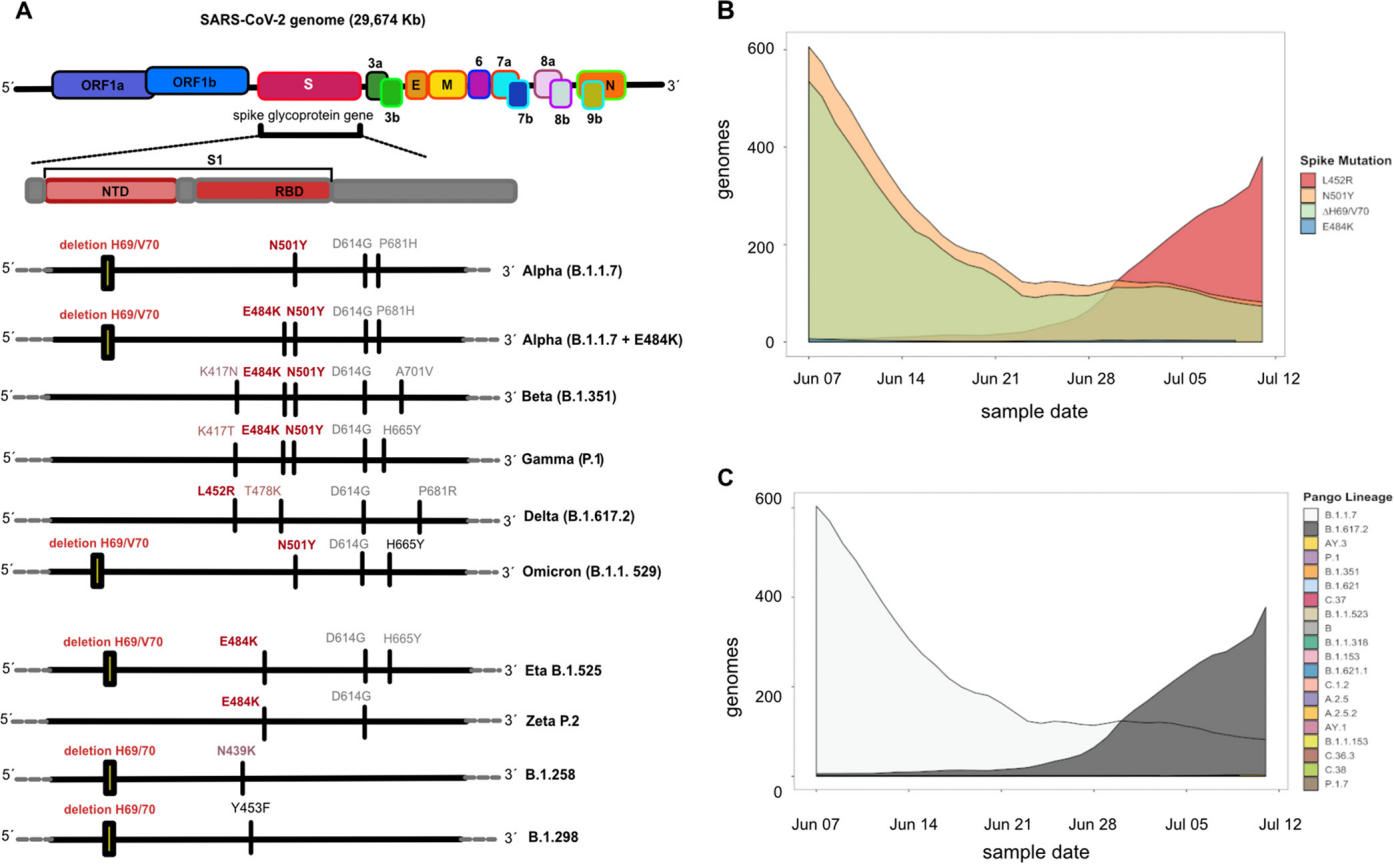
Overview of the key mutations located in the spike glycoprotein during the pandemic and PCR strategies. (A) The spike glycoprotein is located between open reading frame 1b (ORF1b) and ORF3a within the SARS-CoV-2 genome. ΔH69/V70 (2-amino-acid deletion) is located in the N-terminal domain (NTD) of the spike glycoprotein, and the L452R, E484K, and N501Y mutations are located in the receptor-binding domain (RBD). Sets of four variant-specific mutations present in VOCs are indicated. Beta (B.1.351) and Gamma (P.1) as well as the Alpha (B.1.1.7) and Omicron (B.1.1.529) have the same key mutations; therefore, additional mutations should be included in the RT-qPCR. The key mutations are also present in SARS-CoV-2 variants that are not variants of concern and are therefore included in this study for detecting these mutations in patient samples. (B) Prevalence of spike mutations ΔH69/V70, N501Y, E484K, and L452R among SARS-CoV-2 consensus genomes in Denmark between 7 June and 11 July 2021. (C) Composition of variants (by Pangolin nomenclature) harboring key spike mutations.

The severe acute respiratory syndrome coronavirus 2 (SARS-CoV-2) pandemic, which began with the identification and spread of this novel coronavirus in late 2019, has seen the emergence of several virus variants, each with a distinct set of mutations (1). The early detection of new SARS-CoV-2 mutations and associated measures to decrease the risk of spread were important to control local outbreaks of SARS-CoV-2 variants, especially those that have been designated variants of concern (VOCs) (2, 3). The latter are defined by the increased transmissibility and severity of infections and resistance to immunity (4–8). Five variants were reported as VOCs, Alpha (B.1.1.7 and B.1.1.7 plus E484K), Beta (B.1.351), Gamma (P1), Delta (B.1.617.2), and Omicron (B.1.1.529), until the end of 2021 (Fig. 1A to C and Table 1). In these VOCs, combinations of key mutations were present in the spike protein (S) gene: N501Y in the Alpha, Beta, Gamma, and Omicron variants; E484K in the Beta and Gamma variants (9); L452R in the Delta variant, and ΔH69/V70 in the Alpha and Omicron (BA.2) variants.

**TABLE 1:** Overview of SARS-CoV-2 variants and occurrence and evidence of impact

Variant of concern	Variant	Date of first detection (mo and yr), country(ies)	Impact on transmissibility (reference)	Impact on severity (reference[s])	Impact on immunity (reference[s])
Alpha	B.1.117	September 2020, United Kingdom	Yes (5)	Yes (8)	No
Beta	B.1.351	September 2020, South Africa	Yes (27)	Yes (8, 28)	Yes (27, 29)
Gamma	P.1	December 2020, Brazil	Yes (23)	Yes (8)	Yes (7)
Delta	B.1.617.2	December 2020, India	Yes (30)	Yes (6, 30, 31)	Yes (6, 31)
Omicron	B.1.1.529	November 2021, South Africa and Botswana			Yes (32)

The identification of these variants and the mutations defining their mutational signature is largely dependent on whole-genome sequencing (WGS) of SARS-CoV-2 from infected individuals. In addition, WGS of SARS-CoV-2 allows the identification of novel mutations potentially linked to changes in viral properties and/or associated with vaccine breakthrough. However, the advantage of WGS in a pandemic such as coronavirus disease 2019 (COVID-19) also carries with it a significant cost in the forms of reagents, equipment, and turnaround time, with the average time from sample to genome being ~1 to 7 days depending on the scale of sequencing being performed. This has led to the development of alternatives to WGS such as SARSSequencing (SARSSeq), which is based on spike ectodomain sequencing (10) or Sanger sequencing of the spike gene (11).

While such approaches yield cost and reagent savings, the turnaround time, preparation effort, and cost are still higher than those for reverse transcription-quantitative PCR (RT-qPCR) detection platforms. In addition, qPCR technology is arguably one of the cornerstones of modern infectious disease diagnostics; thus, expertise and equipment are readily available and are not hindered by technical issues that might present themselves with newer technologies, which could potentially delay the implementation of such screening approaches. In order to detect SARS-CoV-2 mutations in real time after sample acquisition and to allow implementation on both small and large scales, we developed fast, robust, and flexible RT-qPCRs platforms (also known as variant PCRs) using modified detection probes. Small-scale screening entailed the simultaneous detection of three key mutations in a multiplexed RT-qPCR format, where sets of variant-specific mutations could be replaced by a single signature mutation of concern, for example, the L452R mutation that was present in the Delta variant. The large-scale screening strategy entailed the detection of four key mutations by a combination of multiplexed and single RT-qPCRs running in parallel in a 384-well format. Validation of the large-scale implementation of this RT-qPCR platform was performed for 9,572 positive samples collected between 7 June and 11 July 2021 as part of the national surveillance program in Denmark, using paired WGS consensus genomes derived from SARS-CoV-2-positive samples. The specificity, sensitivity, positive predictive value (PPV), and negative predictive value (NPV) were determined for the large-scale RT-qPCR platform. Both the large- and small-scale platforms were designed to be flexible, where new mutations of concern can be included with ease, and therefore are suitable to support WGS in the surveillance of identified variants of concern.

## RESULTS

### Small-scale screening of SARS-CoV-2 variants of concern.

**FIG 2:**
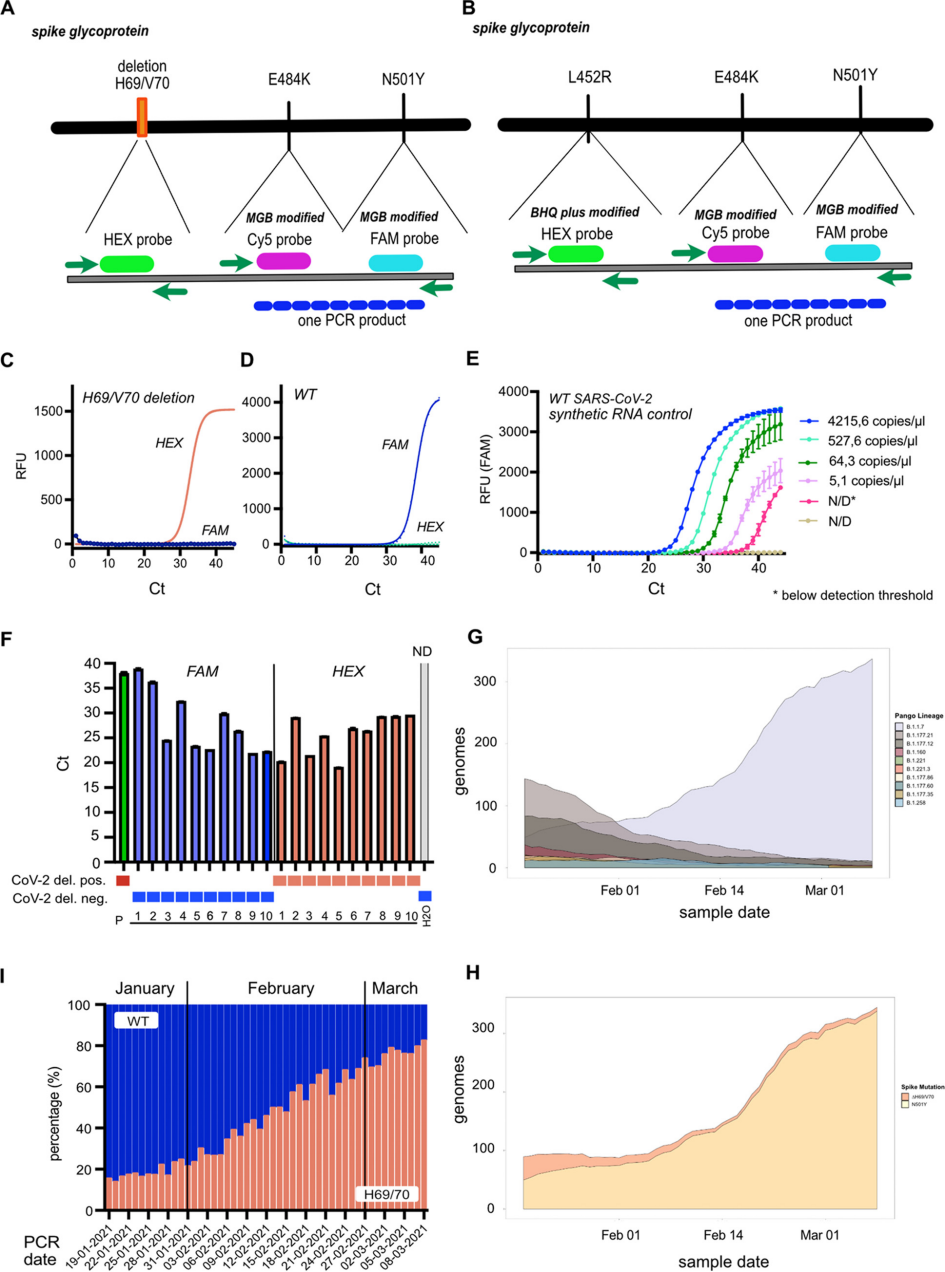
Schematic overview of the PCR platforms and establishment of H69/70 RT-qPCR. (A) Multiplexed RT-qPCR v.1 targeting the ΔH69/70V, E484K, and N501Y mutations. The deletion and the mutations are detected with one probe each, and the E484K and N501Y mutations are detected by one primer pair, resulting in a single amplification product for both mutations. (B) As a proof of concept, ΔH69/70V was replaced with the L452R mutation of the Delta variant (B.1.617.2) in multiplexed RT-qPCR v.2. (C and D) A HEX (6-carboxy-2,4,4,5,7,7-hexachlorofluorescein)-labeled probe detects ΔH69/70 (C), and a 6-carboxyfluorescein (FAM)-labeled probe detects the WT nucleotide sequence (D). (E) Dilution row of the TWIST control (WT SARS-CoV-2) to determine the limit of detection (N/D = not detectable). (F) Detection of ΔH69/V70 (red bars) or the WT sequence (blue bars) in SARS-CoV-2-positive patient samples. The positive control (patient sample with the ΔH69/V70) is displayed as a green bar, and the negative control is displayed as a gray bar. (G) Prevalence of the top 10 SARS-CoV-2 variants in Denmark (based on Pangolin lineage assignments using WGS-derived consensus genomes) from 12 January to 8 March 2021. (I) Large-scale screening of patient samples positive for SARS-CoV-2 by the ΔH69/70 RT-qPCR in the period from 12 January to 8 March 2021. (H) Frequencies of ΔH69/70V and N501Y mutations in Denmark from 12 January to 8 March 2021, as determined from WGS consensus genomes obtained during this period. Mutations are relative to Wuhan-Hu-1/2019 (GenBank accession number MN908947). Error bars in panels E and F indicate the standard errors of the means (SEM) from two technical replicates.

For laboratories with small numbers of positive SARS-CoV-2 samples or without the capacity to screen for SARS-CoV-2 variants on a large scale, we developed a multiplexed RT-qPCR (v.1) that can detect three key mutations (ΔH69/V70, E484K, and N501Y) simultaneously (Fig. 2A). As a proof of concept to determine if a key mutation can be replaced by another, we replaced the ΔH69/V70 mutation with the L452R mutation in multiplex RT-qPCR v.2 (Fig. 2B).

### (i) Multiplexed RT-qPCR v.1.

As the first step in multiplexed RT-qPCR v.1, we developed primer-probe pairs to detect the ΔH69/V70 and wild-type (WT) sequences (Fig. 2C and D). The limit of detection of the ΔH69/V70 RT-qPCR was 5 copies/μL for the ΔH69/V70 mutation, which was determined by performing a dilution serious with a PCR standard TWIST control (Alpha) (Fig. 2E; see also Table S2 in the supplemental material). PCR-positive SARS-CoV-2 patient samples with paired consensus genomes from WGS were included in the ΔH69/V70 RT-qPCR. The ΔH69/V70 or WT nucleotide sequence was detected independently of the amount of SARS-CoV-2 RNA included per sample in the PCR mixture (Fig. 2F) and could be detected in samples of the Alpha variant and the B.1.258 and B.1.1.298 variants. For the later ones, it was known that the ΔH69/V70 mutation was present in these patient samples based on known sequence information (Fig. S1A to C). The ΔH69/V70 RT-qPCR correctly detected ΔH69/V70 in SARS-CoV-2-positive samples and did not amplify samples positive for respiratory tract viruses other than SARS-CoV-2 (Table S3). After successful validation, this RT-qPCR was incorporated as a part of the national surveillance program in Denmark, in a large-scale screen for SARS-CoV-2 variants harboring ΔH69/V70 (starting in December 2020). By mid-February 2021, the Alpha variant was the most prominent variant in Denmark (Fig. 2G), and at the end of March, about 80% of all SARS-CoV-2 patient samples tested positive for ΔH69/V70 (Fig. 2I), which was confirmed by WGS (Fig. 2H; Fig. S1D). While the Alpha variant was the most dominant variant at that time, the Beta and Gamma variants were still circulating in Denmark (Fig. S1D). Therefore, we sought to determine if the ΔH69/V70 RT-qPCR could be multiplexed, which would then allow the incorporation of further mutations present in these other VOCs. Running the ΔH69/V70 RT-qPCR together with the diagnostic SARS-CoV-2 E-Sarbeco PCR (E gene) (12) showed that the sensitivity of this PCR was not reduced when multiplexed with the E-Sarbeco RT-qPCR (Fig. S1E), suggesting its insensitivity to multiplexing. Primers and probes were developed to detect the key L452R, E484K, and N501Y mutations for the other VOCs. Compared to ΔH69/V70, where the probe targets a stretch of a deletion of 6 nucleotides, the probes for the three key mutations listed above differ by only 1 nucleotide substitution within the S gene. Therefore, the affinity of the binding of these probes to the mutations or the WT sequence was increased by either black hole quencher plus (BHQplus), locked nucleic acid (LNA), or minor groove binding (MGB) conjugation. All primer-probe combinations with all three probe modifications were tested for each mutation. It was observed that MGB-conjugated probes for the N501Y mutation were superior to LNA-conjugated probes, where a specific signal was detected for either the mutation or the WT sequence. In contrast, for LNA-conjugated probes in the N501Y RT-qPCR, additional allelic discrimination analysis was needed to discriminate the intensity of the signal for the mutation or the WT probe at a threshold cycle (*C_T_*) value of 45 (Fig. S2A to D).

**FIG 3:**
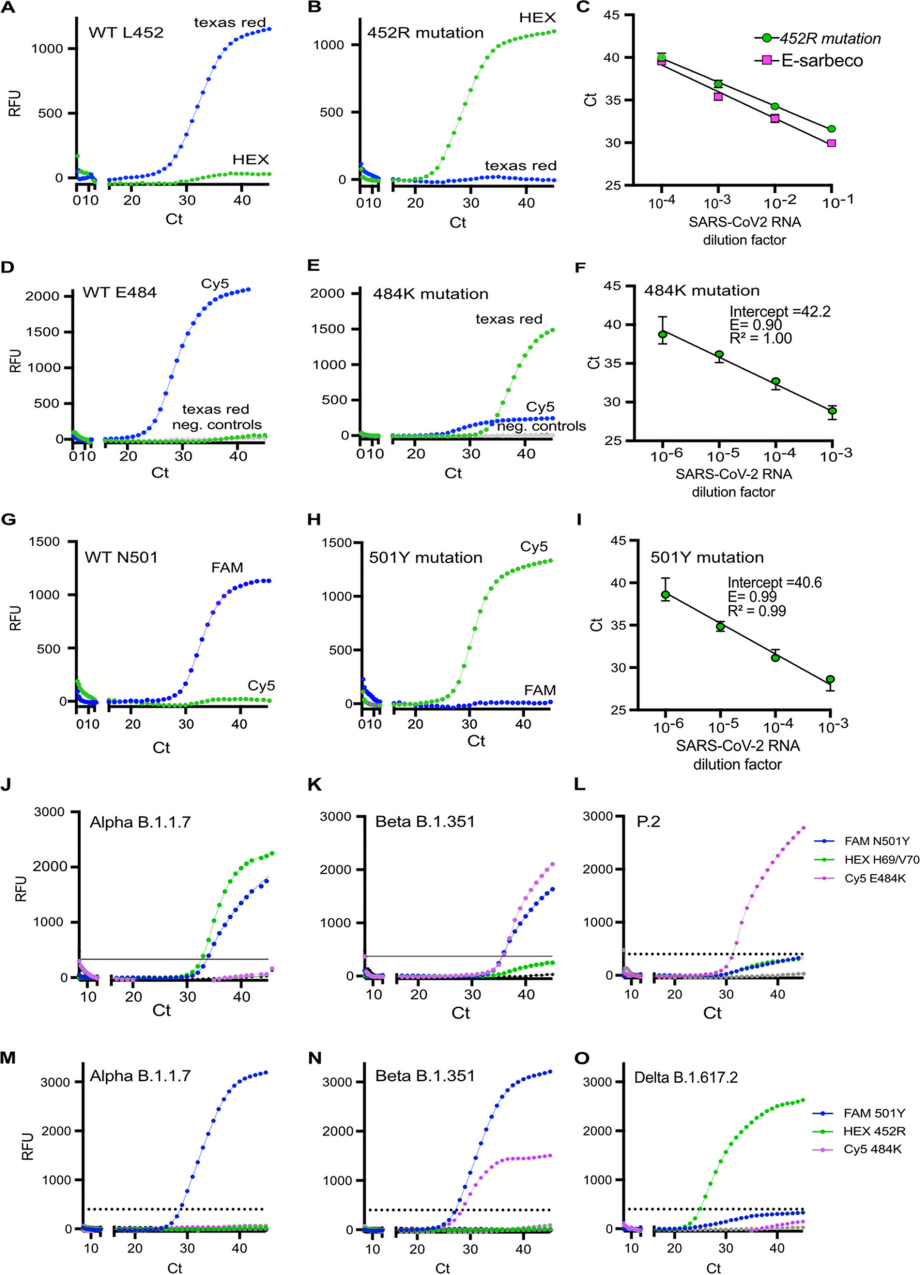
Performance of primers and probes for the L452R, E484K, and N501Y mutations and screening of patient samples positive for SARS-CoV-2 with different mutations of concern present in multiplexed RT-qPCR v.1 and v.2. (A and B) BHQplus-conjugated probes detecting the L452 WT SARS-CoV-2 nucleotide sequence and the 452R mutation. (C) Dilution row of a patient sample with known whole-genome sequence information for the Delta variant (B.1.617.2) tested in parallel by the L452R and E-Sarbeco RT-qPCRs. (D) MGB-conjugated probes detecting the E484 WT SARS-CoV-2 nucleotide sequence and the 484K mutation. (F) Dilution row of the TWIST control (Gamma P.1) included in the E484K RT-qPCR. (G) MGB-conjugated probe detecting the N501 WT SARS-CoV-2 nucleotide sequence and the Y501 mutation. (I) Dilution row of the TWIST control (Alpha B.1.1.7) included in the N501Y RT-qPCR. (J to L) Detection of three key mutations, ΔH69/V70, E484K, and N501Y in patient samples with known whole-genome sequence information identified as Alpha (B.1.1.7), Beta (B.1.351), and P.2 variants by multiplexed RT-qPCR v.1. (M to O) Detection of three key mutations, L452R, E484K, and N501Y, in patient samples with known whole-genome sequence information identified as Alpha (B.1.1.7), Beta (B.1.351), and Delta (B.1.617.2) variants by multiplexed RT-qPCR v.2. Error bars in panels C, F, and I indicate the SEM from two technical replicates.

BHQplus-conjugated probes were found to be very specific for the L452R mutation compared to the LNA- and MGB-conjugated probes (Fig. 3A and B). The limit of detection for L452R was determined by a dilution series of a patient sample with known sequence information for the Delta variant and tested in parallel in the L452R RT-qPCR and the E-Sarbeco RT-qPCR (Fig. 3C). The L452R RT-qPCR was about 2-fold less sensitive than the E-Sarbeco RT-qPCR (Fig. 3C).

The best results for the E484K and N510Y mutations were gained using MGB-conjugated probes that were refined to generate a signal specific for the mutation or the WT nucleotide (Fig. 3D, E, G, and H). By performing a dilution series with the TWIST control for the Beta and Gamma variants, the limits of detection for the E484K and N501Y RT-qPCRs were found to be 52 and 5 copies/μL, respectively (Fig. 3F and I; Table S1).

As the signal detected was specific for either the key mutations or the WT sequence, it was possible to include only the probes detecting the key mutations (L452R, E484K, and N501Y) or ΔH69/V70 in multiplexed RT-qPCR v.1 and v.2 (Fig. 3J to O). The probe for ΔH69/70 was further modified as a Zen-conjugated probe in multiplexed RT-qPCR v.1 to increase the signal intensity for this probe competing with the MGB-conjugated probes for the E484K and N501Y mutations. Testing SARS-CoV-2-positive patient samples with known whole-genome sequence information by the multiplexed RT-qPCR (v.1), the key mutations ΔH69/V70, E484K, and N501Y, if present in the Alpha, Beta, B.1.5125, and P.2 variants, were detected simultaneously in all patient samples (Fig. 3J to L; Table S4). As the mutations were detected by a single probe, a threshold was set to differentiate between specific and unspecific signals (Fig. 3J to O). The limit of detection for multiplexed RT-qPCR v.1 was moderately reduced to around 50 copies/μL for the different mutations, compared to around 5 copies/μL for the single RT-qPCRs (Tables S1 and S6). To determine the specificity of RT-qPCR v.1, we tested samples containing respiratory tract viruses other than SARS-CoV-2. Five positive signals could be detected for samples of respiratory tract viruses but with a *C_T_* value of >38 in multiplexed RT-qPCR v.1 (Table S5). Repeating the experiments twice with the same samples in RT-qPCR v.1 resulted in negative results (Table S5).

### (ii) Multiplexed RT-qPCR v.2.

In order to investigate the robustness of the multiplexed RT-PCR, we investigated if ΔH69/V70 could be replaced by the L452R mutation in multiplexed RT-qPCR v.2. As it is recommended to limit the number of MGB-conjugated probes in multiplex RT-qPCR, we combined the two MGB-conjugated probes for the E484K and N501Y mutations with a BHQplus-conjugated probe for the L452R mutation. With this approach, the three key mutations L452R, E484K, and N501Y could be simultaneously detected in all samples with known sequence information for the Alpha, Beta, and Zeta variants in multiplexed RT-qPCR v.2 (Table S3).

The multiplexed small-scale RT-qPCR platform offers a flexible and fast detection system to rapidly identify key mutations present in SARS-CoV-2 VOCs and mutations of interest. Notably, new key mutations can be accommodated by exchanging one of the existing sets.

### Large-scale screening by variant RT-qPCR as part of the national surveillance program in Denmark.

**FIG 4:**
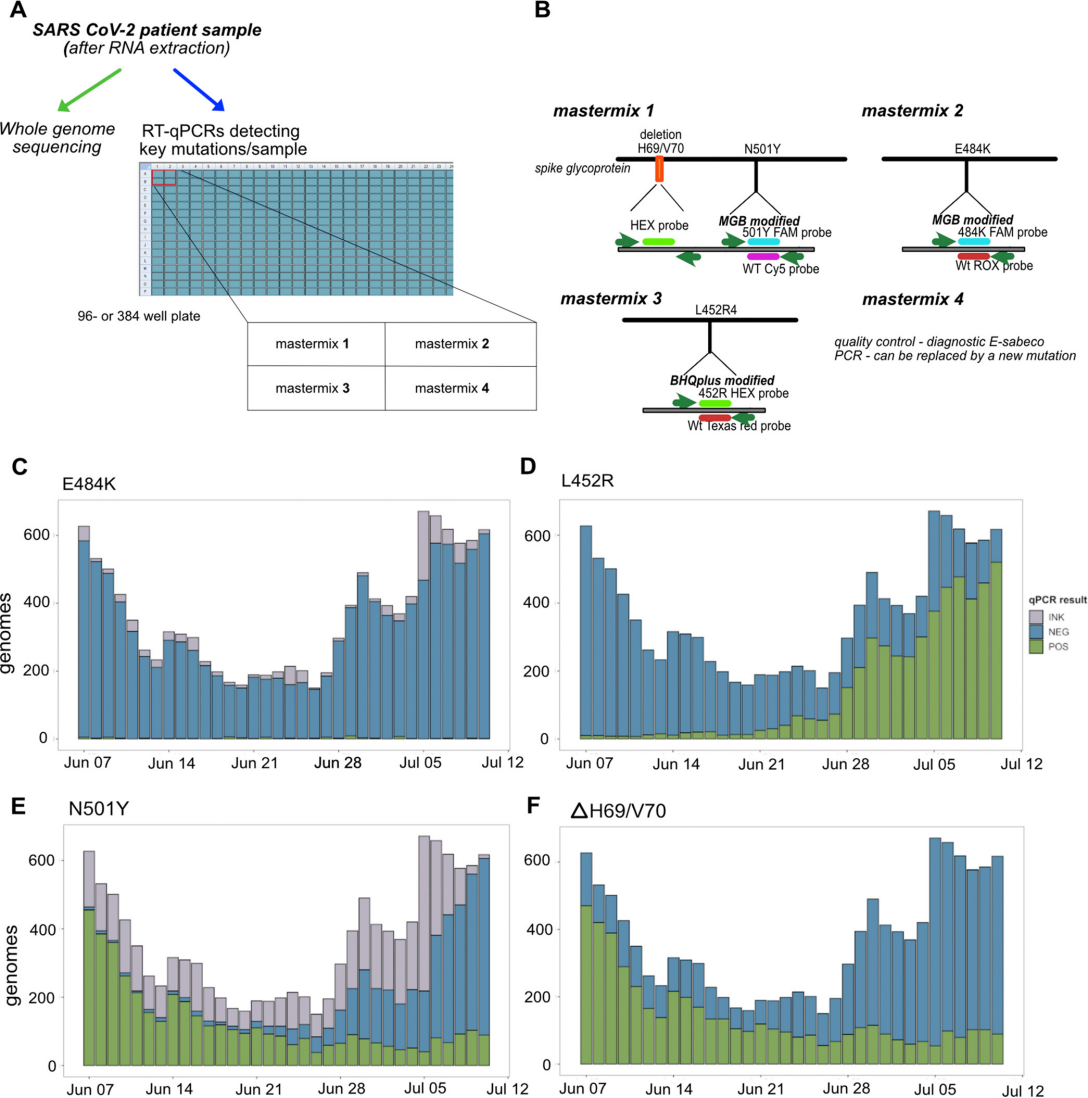
Large-scale screening for four key mutations. (A) Schematic overview of large-scale screening. (B) Primers and probes included in the multiplexed and single PCRs running in the 384-well-plate format. (C to F) RT-qPCR results from large-scale screening for each target mutation, E484K (C), L452R (D), N501Y (E), and ΔH69/70V (F), shown as positive (POS), negative (NEG), or inconclusive (INK).

The same primers and probes designed for the four key mutations included in the multiplexed RT-qPCR for small-scale screening were further validated for large-scale screening to support the national surveillance program in Denmark, in addition to WGS. Large-scale screening consisted of RT-qPCRs running in parallel on a 384-well plate, allowing the parallel detection of the four key mutations (Fig. 4A and B). The two key mutations ΔH69/V70 and N501Y that ran as a multiplexed RT-qPCR on a large scale were detected, if present, in 17/17 patient samples (100%) with known sequence information for the Alpha and Beta variants (Table S7). The L452R and E484K mutations were correctly detected, if present, in 18/18 (100%) and 31/31 (100%) patient samples with known sequence information for the Alpha, Beta, Delta, Zeta, or B.1.525 variant in single RT-qPCRs (Tables S8 and S9). The ΔH69/V70/N501Y, L452R, and E484K RT-qPCRs for large-scale screening were specific, as these did not yield positive signals in samples positive for common respiratory tract viruses other than SARS-CoV-2 (Table S2). Based on these results, the RT-qPCRs were implemented in the large-scale screening strategy in place at TestCenter Denmark, where the sensitivity and specificity were also validated using paired consensus genomes (Fig. 4C to F).

To validate the large-scale RT-qPCR screen, results from 9,572 samples positive by both RT-qPCR and WGS over a 5-week period from 7 June to 11 July 2021 were compared. This period was selected due to the presence of all four key mutations of interest in genomes sequenced as part of the Danish national surveillance strategy. It was also during this period that the dominant Alpha SARS-CoV-2 variant (13) was seen to be replaced by the more transmissible Delta variant (14) in Denmark. This then allowed a rigorous test of the RT-qPCR strategy due to the presence and absence of these key mutations among these multiple variants (Fig. 1A and B). A daily range of 150 to 671 samples were analyzed by multiplex RT-qPCR during this period, and the results were characterized as either positive or negative for a given key mutation (Fig. 4C to F). It was observed that there was a small number of inconclusive results (*C_T_* values of between 38 and 40 or end relative fluorescence unit (RFU) value of <200 at a *C_T_* value of 45) among the E484K RT-qPCRs as well as the N501Y RT-qPCR, which could be attributed to probe manufacturing issues beyond our control; the replacement of the probes resulted in a significant reduction in the number of such results from this reaction (Fig. 4E, 10 to 11 July 2021). This probe was found to be more sensitive to the concentrations of the samples; thus, samples with high *C_T_* values in the initial E-Sarbeco-based analysis tended to yield inconclusive results. Thus, the N501Y RT-qPCR was more sensitive to minor variations in batch quality. In order to validate all RT-qPCR results and determine the specificity and sensitivity of these primer-probe combinations, WGS consensus genomes from the same samples were used as a reference standard.

**FIG 5:**
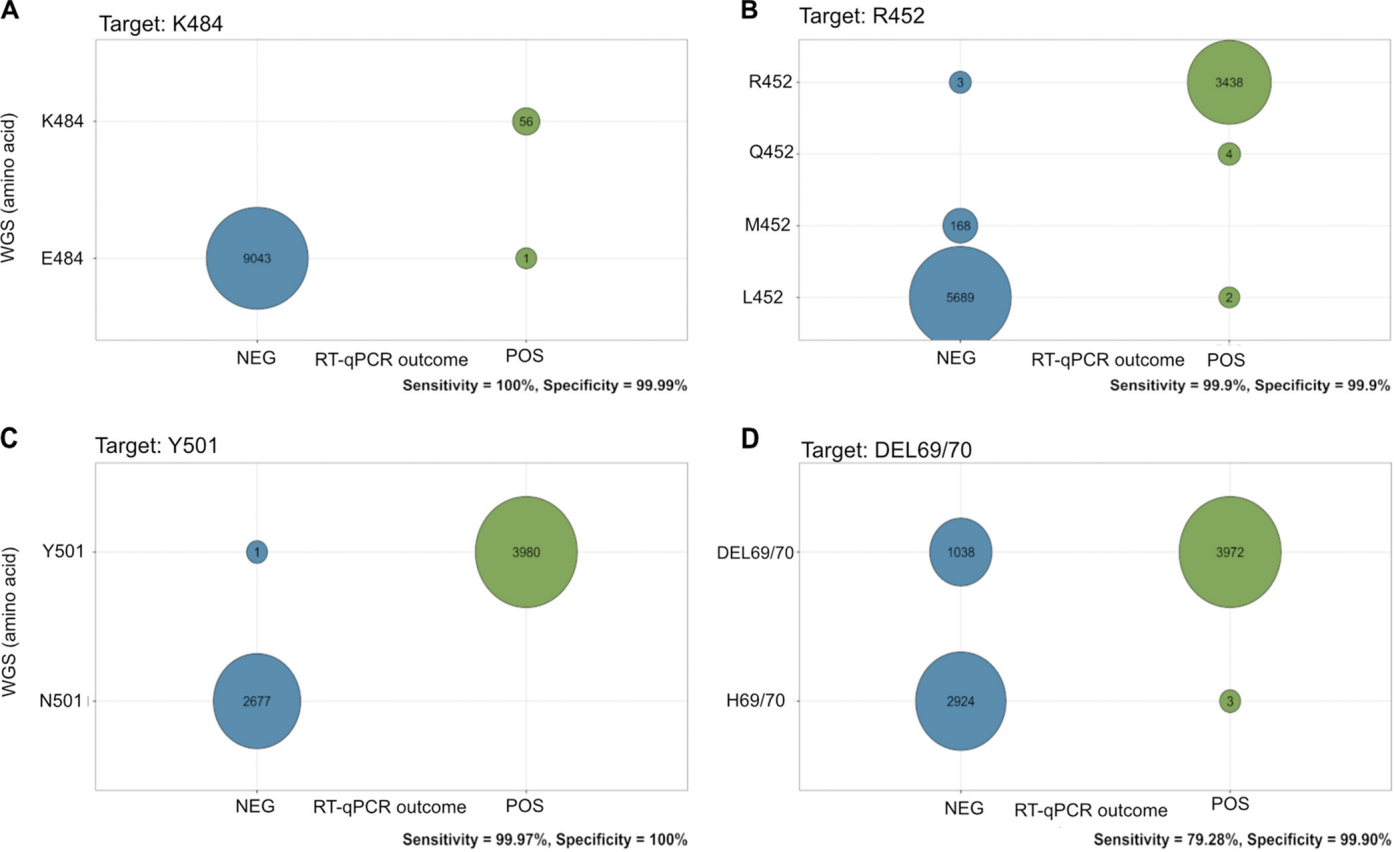
Validation of RT-qPCR results from large-scale screening. The concordance between RT-qPCR results and WGS results is represented as graphical matrices, with each cell represented as a circle showing the number of samples that correspond to a positive (POS) (green) or negative (NEG) (blue) RT-qPCR result (horizontal axis) and a given amino acid derived from WGS consensus genomes (vertical axis) for E484K (A), L452R (B), N501Y (C), and ΔH69/70V (D). The sensitivity and specificity of each RT-qPCR are shown on the bottom right of each panel.

WGS was performed on all positive samples during the study period using the ARCTIC Network’s PCR scheme v3 (see Materials and Methods), and the aligned S gene sequences from the resulting consensus genomes were used to validate the results of each of the three RT-qPCRs by comparison of the translated codons to the RT-qPCR results for each position encoding the four key mutations of interest in this study. Validation was performed on samples where both a valid RT-qPCR result and a consensus genome sequence were obtained, the numbers of which differed for each of the four key mutations for various technical reasons anticipated at this scale (see above). The validation results (Fig. 5) showed good agreement between amino acids translated from WGS and RT-qPCR results for E484K, N501Y, and L452R (Fig. 5A to C). The determination of concordance proved to be less straightforward for ΔH69/V70 due to the poor coverage of the deletion among the generated consensus genomes, resulting in a discordant fraction between the deletion and negative RT-qPCR results (Fig. 5D). It was also observed among the consensus genomes used for this validation that amino acid 452 in the spike protein was more mutable than the other positions that form this set of key mutations, with L, R, M, and Q being observed at this position depending on the lineage (Q484 was not observed in genomes during the selected period but has been recorded in global surveillance data).

In order to meaningfully compare and describe the relative performance of the RT-qPCR strategy from the results of the large-scale screen as well as to determine the rates of true-positive or true-negative results of these combinations of primers and probes, the specificity and sensitivity of each primer-probe combination were determined using established methods used to characterize diagnostic testing (15). Using the validation of the RT-qPCR with WGS as a reference standard, the specificities and sensitivities were calculated for all primers and probes for the four key mutations, and it was observed that all four RT-qPCRs were highly specific (>99.9%), and three out of four assays were highly sensitive (>99.9%) (Fig. 5A to D). The sensitivity of the probe for the detection of ΔH69/V70 was observed to be reduced (79.28%) due to the significant number of deletions by WGS, which were assayed to be negative by RT-qPCR; however, given the challenge with read alignments around genomic regions containing insertions or deletions, this was postulated to be largely due to the determination of the deletion in WGS consensus genomes. In addition to the specificity and sensitivity of the primer-probe combinations, the positive predictive value (PPV) and negative predictive value (NPV) of these combinations were also determined, which indicate the ability of a diagnostic assay or test to accurately detect a condition or, in this case, a mutation (15). The determination of the PPV and NPV takes into account the specificity and sensitivity of the primer-probe combinations as well as the prevalence of the four key mutations among the sequenced SARS-CoV-2 genomes during the study period. It was determined that all four primer-probe combinations had a PPV of at least 97%, and three out of four had an NPV of 99.9%, with the ΔH69/V70 assay having an NPV of 75.43% (Fig. 5A to D). From the results of the large-scale screening, it can be seen, therefore, that the specificity and sensitivity as well as the PPV and NPV all point toward the viability of this RT-qPCR strategy in a large-scale diagnostic setting (Table 2).

**TABLE 2:** Positive and negative predictive values for all 4 RT-qPCR assays during the period of large-scale screening (7 June to 11 July 2021)^*a*^

Mutation	Prevalence (%) (no. of samples)	PPV (%)	NPV (%)
ΔH69/V70	61.1 (5,010)	99.9	75.4
N501Y	52.3 (5,846)	100.0	99.9
E484K	0.6 (60)	97.7	100.0
L452R	35.9 (3,441)	99.8	99.9

a Mutation prevalence estimates were calculated based on consensus sequences from 9,572 positive samples (as determined by E-Sarbeco PCR) obtained during this period.

In summary, we developed an RT-qPCR system for the large-scale screening of four key mutations in parallel that is highly specific and sensitive, validated by a comparison of the qPCR and WGS data for 9,572 samples that were tested in parallel.

## DISCUSSION

RT-qPCR is a fast, standard method for SARS-CoV-2 detection and was established at the start of the pandemic in January 2020 (12). RT-qPCR platforms for small- and large-scale screening can support the detection of mutations of concern present in SARS-CoV-2 variants. This is of special interest for countries lacking or with smaller-scale infrastructure for large-scale WGS sequencing, the gold standard for SARS-CoV-2 variant surveillance. Here, a detection system is needed that is fast, robust, and flexible and that enables the detection of known diagnostic mutations almost in real time after sample collection, as we show in this study. Here, we describe validated and advanced RT-qPCR platforms for small- and large-scale screening that can simultaneously detect mutations of concern within the S gene of SARS-CoV-2, with a short turnaround time for large-scale screening of 12 to 24 h to reporting to the public health system. These RT-qPCR platforms can be established quickly, and new mutations can be detected rapidly, an important advantage for monitoring the course of a pandemic. Troubleshooting is possible without extensive knowledge, and platforms can be adjusted to the existing infrastructure of the laboratory for large-scale screening and data evaluation. Moreover, these RT-qPCR platforms carry a low cost of about 10 Danish kroner (2 U.S. dollars) per reaction and can therefore be established and adapted in countries without the resources for large-scale WGS surveillance.

The standardized protocol for SARS-CoV-2 RT-qPCR makes it easy to implement in diagnostic laboratories worldwide, where the equipment needed is commonly present. This could be advantageous compared to new methods such as reverse transcription–loop-mediated isothermal amplification (RT-LAMP) or CRISPR, which have been described for SARS-CoV-2 detection, delivering fast test results and being applied without extensive laboratory equipment (16–18).

Currently, there are only limited studies on RT-LAMP for commercial point-of-care testing (3). Moreover, CRISPR is still in its early stages of development (19) and has been shown to be less sensitive than RT-qPCRs (3), which are still the methods of choice for most diagnostic laboratories. Based on recent advances in the modifications of conjugated probes, RT-qPCRs can be designed to detect mutations within the SARS-CoV-2 genome consisting of a single nucleotide polymorphism (SNP).

For small-scale screening, the multiplexed RT-qPCR was developed using Luna probe one-step RT-qPCR mix, which offers the possibility of increasing the input template concentration for amplification targets with a low RNA concentration, as it is four times more concentrated. However, this was not an advantage when establishing multiplexed RT-qPCR v.1 and v.2, as SARS-CoV-2 RNA concentrations vary among patient samples, leading to artificial signals. In contrast, adjusting the primer and probe concentrations for each mutation resulted in the highly specific and sensitive detection of the corresponding mutations present in the different SARS-CoV-2 variants (Fig. 3J to O; see also Table S4 in the supplemental material). Moreover, by reducing the number of probes in the multiplexed RT-qPCR, we could maintain a sensitive system for diagnostic use by including one probe for each mutation. This was possible by designing and testing combinations of primers and MGB-, LNA-, or BHQplus-conjugated probes that yield specific signals for the mutant and WT sequences. So far, only mutations of concern within the S gene have been included in the multiplexed RT-qPCR platform for small-scale screening, but running the ΔH69/70 RT-qPCR as a multiplexed PCR together with the clinical E-Sarbeco RT-qPCR did not reduce the sensitivity of the PCR (Fig. 2E). As up to five targets can be included in the multiplex RT-qPCR using the Luna probe one-step RT-qPCR mix, additional targets located in different loci of the SARS-CoV-2 genome other than S could be incorporated into the proposed platform.

Large-scale RT-qPCR screening of mutations present in VOCs required that a number of technical and analytical considerations be fulfilled: (i) the RT-qPCR must be highly specific and sensitive to minimize or avoid false-positive results, (ii) it should be of sufficient robustness to allow the massive scalability required for a pandemic, (iii) it must not interfere with diagnostic PCR to detect SARS-CoV-2 to reduce the risk of potential PCR contamination, and (iv) an advanced, automated evaluation system is needed to detect erroneous results. It was found that the large-scale RT-qPCR platform met the technical and analytical considerations outlined above. The current design is based on sample preparation in a 96-well format and subsequent RT-qPCR in a 384-well format. This allows each sample to be analyzed by four separate sets of primers and probes, which enables the analysis of 4 mutations for up to 92 samples and 4 controls (1 negative and 3 positive) in parallel in a single run. The system is flexible, as the combination of target mutations can be adjusted over time according to current needs.

From the large-scale screen, it was determined that the RT-qPCR platform described in this study is generally of very high specificity and sensitivity and performs well in terms of its PPV and NPV, indicating its utility in such large-scale diagnostic screens. The period for large-scale screening and validation was specifically chosen to interrogate the robustness of this system in a pandemic transition period with ongoing lineage replacement; such a period involves the waning of certain variants such as the Alpha variant and its signature mutations ΔH69/V70 and N501Y along with the rise of a different variant like Delta with a different signature mutation (L452R), as was seen in June 2022. In order to have diagnostic value, surveillance mechanisms to track these exclusive signatures, and which do not yield full genomes, must have adequate sensitivity and specificity to adequately distinguish between such signature mutations. In this respect, the sensitivity and specificity of our system were excellent, falling short in sensitivity in only one assay (ΔH69/V70) due to distinct technical issues that revolve around the WGS reference standard and not the RT-qPCR itself. First, the challenge of read alignment around genome deletions leads to ambiguous base calls around these regions. Second, in large-scale amplicon-based genomic surveillance, dropouts are not a rare occurrence, and a certain degree of N counts is therefore considered permissible (typically less than 5 to 10% of the consensus genome). Tracts of N’s around this region were observed around the deletion, and this was largely responsible for the challenges in identifying a deletion from WGS consensus genomes. However, this was not the case for SNPs leading to nonsynonymous substitutions as seen with N501Y, E484K, and L452R. Interesting insights into the specificity and the sensitivity of the RT-qPCR system were also observed with the results around the L452R mutation, given that position L452 in the spike protein exhibited more than a single amino acid change during the pandemic and, indeed, the time frame of the large-scale screening performed. The validation showed that all samples with L452Q in the spike protein recorded a positive result, whereas those with L452M recorded exclusively negative results. Given that the codon observed from WGS encoding Q was CAG, and the corresponding codon encoding L at the same position was CGG, this was unsurprising, given that it is impossible to preempt mutations. In addition, the ATG codon, which is more distant from CGG and which encodes M at this position, was not detected by the L452R-specific probe, which alludes to the specificity and sensitivity of the RT-qPCR probe at position 452. This was also highly important for the detection of the emerging Omicron variant at the beginning of December 2021 in Denmark, with the probe detecting the L452 WT sequence in a background of 100% Delta (given L452R prevalences of 97.1% globally and about 98% in Denmark).

One of the major technical developments for control seen in this pandemic is the early detection of new VOCs to gain time to characterize the increased infectivity potential borne of vaccine failure. The screening and isolation of individuals are therefore time sensitive and require a rapid turnaround. While WGS of positive samples allows the accurate identification of these variants or mutations to enable their tracking and control, this requires a longer turnaround time and higher costs in terms of reagents, equipment, and expertise. The use of RT-qPCR systems such as the one described in this study allows the rapid identification of mutations of concern, which in turn enables the near-real-time tracking of these mutations and, correspondingly, rapid decision-making about testing, contact tracing, and isolation. This enabled the rapid reaction of the public health system in Denmark to the detection of VOCs, with the added benefit of gaining time to implement its vaccination schedule. This was in line with modeling showing that minimizing testing delays had the largest impact on reducing onward transmissions (20). The data generated also helped in evaluating the severity of a new SARS-CoV-2 variant, for example, by evaluating the risk of hospitalization after Omicron infection (21).

The flexibility of the RT-qPCR platform makes it ideal for the rapid detection of mutations of concern, as we show here in our study. This has been proven to be essential for the detection of new VOCs, and the RT-PCR platforms can be extended to include novel VOCs. The current shift in our consideration of the pandemic (toward endemicity) suggests that such monitoring and screening might have to continue for a considerably longer time, making this system extremely viable in the long term and, indeed, in future outbreaks and pandemics.

## MATERIALS AND METHODS

### Ethics.

Exemption for review by the ethical committee system and informed consent were given by the Committee on Biomedical Research Ethics—Capital Region in accordance with Danish law on assay development projects.

### Virus isolation.

SARS-CoV-2 isolates representative of VOCs (Delta variant B.1.617.2, Alpha variant B.1.1.7, and Beta variant B.1.351) were isolated from PCR-positive throat swabs collected in phosphate-buffered saline (PBS) from community testing facilities (TestCenter Denmark) and BioBank Denmark, which form part of the Danish national surveillance program (4). Primary isolation was performed in 24-well culture plates with 5 × 10^4^ Vero E6 cells/well seeded the day before. Cells were washed once with PBS, and 150 to 250 μL of swab material and 150 to 250 μL of infection medium (Dulbecco’s modified Eagle medium [DMEM] with 1% penicillin-streptomycin) were added to each well. After 1 h of incubation at 37°C with 5% CO_2_, 1 mL/well of propagation medium (DMEM with 1% penicillin-streptomycin and 5% fetal calf serum) was added, and the cultures were further incubated until cytopathic effect (CPE) was observed. Isolations performed later during the pandemic additionally used 1.5 μg/mL amphotericin B in the propagation medium. All cell culture reagents were obtained from Gibco, Thermo Fisher Scientific, Waltham, MA. Upon the observation of CPE, the supernatants were aliquoted and frozen at −80°C. Subsequent passages to expand virus stocks were performed in 75-cm^2^ flasks seeded with 1.5 × 10^6^ Vero E6 cells the day before. Twenty-five microliters of the primary isolate supernatant was used as the inoculum in the presence of 2 mL of infection medium. After incubation for 1 h at 37°C with 5% CO_2_, flasks were supplemented with 10 mL of propagation medium (without amphotericin) and incubated until CPE was observed. The supernatants were then clarified by centrifugation for 5 min at 300 × *g* and stored as single-use aliquots at −80°C.

### RT-qPCR standards and patient samples used for validation of the small- and large-scale platforms.

The following controls and patient samples were included in the RT-qPCRs.

Diagnostic samples positive for common respiratory pathogens, including human coronaviruses 229E, HKU1, NL63, and OC43, as well as adenovirus and rhinovirus, were obtained as extracted nucleic acids from the human diagnostic virus PCR laboratory at Statens Serum Institute, Denmark.

Extracted influenza virus RNAs from viruses cultured in Madin-Darby canine kidney (MDCK) cells (A/Christchurch/16/2010 [H1N1], pdm09-like virus, B/Phuket/3073/2013-like virus, and B/Brisbane/60/2008-like virus) were provided by the WHO Collaborating Centre for Reference and Research on Influenza, The Francis Crick Institute, London, United Kingdom. For all non-SARS-CoV-2 controls, a high concentration (*C_T_* of <30) was determined by qPCR before they were implemented as controls to prove specificity.

TWIST synthetic SARS-CoV-2 controls (MT007544.1/Australia/VIC01/2020, MT103907/England/205041766/2020, MT104043/South African/KRISP-EC-K005299/2020, and MT104044/Japan/IC-0564/2021) were bought from TWIST Bioscience and used as PCR standards for the WT and the Alpha (B.1.17), Beta (B.1.351), and Gamma (P.1) variants, respectively.

SARS-CoV-2 controls that present the key mutations L452R, E484K, N501Y, and ΔH69/V70 were obtained from extracted virus cultures (described above) and diluted in DNase/RNase-free water to generate *C_T_* values of between 25 and 30 in the subsequent RT-qPCR. The virus cultures were heat inactivated (56°C for 45 min) before use.

For the large-scale RT-qPCR platform, the positive and negative controls were already included in the extraction step before performing the RT-qPCR. Therefore, as a negative control, 1× Dulbecco’s Phosphate Buffered Saline (PBMS) (pH 7.2) was used, in contrast to the UltraPure water used as the negative control in the small-scale platform.

SARS-CoV-2-positive patient samples were obtained from the Danish National BioBank.

### Nucleic acid extraction.

For the small-scale RT-qPCR platform, total nucleic acid from SARS-CoV-2-positive patient samples was extracted with the MagNA Pure96 extraction robot using the MagNA Pure 96 DNA kit as well as the viral NA small-volume (SV) kit. Extraction was performed according to the viral NA plasma SV protocol with 200-μL input and 100-μL elution volumes.

For the SARS-CoV-2 positive controls (extracted viruses), 120 μL of the supernatant from SARS-CoV-2-infected cells was mixed with 120 μL of MagNA Pure lysis buffer (Roche) and extracted as described above for the SARS-CoV-2-positive patient samples.

For the large-scale RT-qPCR platform, RNA from SARS-CoV-2 patient sample screening was extracted with the Beckman Coulter Biomek i7 robot using the Beckman Coulter RNAdvance whole-blood kit with 200-μL input and 50-μL elution volumes. The extracted RNA for all sample types was stored at −80°C until use.

### Primer and probe design.

SARS-CoV-2 sequences were retrieved from positive patient samples identified through the national surveillance program in Denmark. Sequences were aligned, and based on the alignment, primers and probes were designed using Geneious Prime 2021.0.

Two probes were designed for each key mutation: one to detect the wild-type (WT) nucleotide sequence and one to detect the mutation. The probe design was refined to detect the key mutations (L452R, E484K, N501Y, and Δ69/V70) with only one probe in the multiplex RT-qPCRs. To ensure stable allelic discrimination analysis, probes to detect the mutations with only 1 nucleotide exchange were either MGB, LNA, or BHQplus modified, which increased the melting temperature (*T_m_*) of the probes. The calculation of the *T_m_* of the MGB probes was adapted from methods described previously (22).

The primers and probes listed in Table 3 were synthesized by Biosearch Technologies, Denmark, except for the MGB-conjugated probes, which were synthesized by Eurogentec, Belgium, and the Zen-conjugated probe, which was synthesized by Integrated DNA Technologies, Belgium. All oligonucleotides were purified by high-performance liquid chromatography (HPLC).

**TABLE 3:** Primer and probe sequences^*a*^

Purpose and target	Primer or probe name	*T_m_* (°C)	Primer or probe sequence (5′–3′)	Vol (μL)^*c*^	Master mix ID(s)
SARS-CoV-2 primary diagnostic assay					
E gene	E_Sarbeco^31^_forward	58.8	ACAGGTACGTTAATAGTTAATAGCGT	0.1	
	E_Sarbeco_reverse	61.0	ATATTGCAGCAGTACGCACACA	0.1	
	E_Sarbeco_probe1	66.3	FAM-ACACTAGCCATCCTTACTGCGCTTCG-BHQ1	0.05	

Primers and probes used in small- and large-scale testing for key mutations					
ΔH69/V70	SARS-CoV-2_ΔH69/V70 forward	58.5	ACATTCAACTCAGGACTTGTTCT	0.1	1, 2, 5
	SARS-CoV-2_ΔH69/V70 reverse	58.0	TCATTAAATGGTAGGACAGGGTT	0.1	1, 2, 5
	SARS-CoV-2_ΔH69/V70 probe^*b*^	61.2	HEX-TTCCATGCTATCTCTGGGACCA-BHQ2	0.05	1, 2, 5
N501Y	SARS-CoV-2 N501Y forward	57.7	TGTTACTTTCCTTTACAATCATATGGT	0.1	1, 2, 5, 6
	SARS-CoV-2 N501Y reverse	58.9	TGCTGGTGCATGTAGAAGTTCA	0.1	1, 2, 5, 6
	SARS-CoV-2 501Y_mutant MGB probe	64.8	FAM-CCCACT**T**ATGGTGTTGGT-MGB	0.05	2, 5, 6
	SARS-CoV-2 N501 WT MGB probe	64.8	Cy5-CCCACT**A**ATGGTGTTGGT-MGB	0.05	2
E484K	SARS-CoV-2_E484K forward	58.5	AGGAAGTCTAATCTCAAACCTTTTGA	0.1	3, 5, 6
	SARS-CoV-2_E484K reverse	60.2	GTCCACAAACAGTTGCTGGTG	0.1	3, 5, 6
	SARS-CoV-2_484K_mutant MGB FAM probe	64.6	FAM-TGGTGTT**A**AAGGTTTTAAT-MGB	0.05	3
	SARS-CoV-2_E484K_WT MGB probe	63.5	Texas Red-TGGTGTT**G**AAGGTTTTAA-MGB	0.05	3
L452R	SARS-CoV-2_L452R forward	60.5	CAGGCTGCGTTATAGCTTGGA	0.1	4, 6
	SARS-CoV-2_L452R reverse	57.1	CCGGCCTGATAGATTTCAGT	0.1	4, 6
	SARS-CoV-2_452R_mutant BHQ+ probe	58.2	HEX-TATAATTACC**G**GTATAGATTGTT-BHQ1	0.05	4, 6
	SARS-CoV-2_L452_WT BHQ+ probe	58.0	Cal Fluor Red 610-TATAATTACC**T**GTATAGATTGTTTA-BHQ2	0.05	4
	
Probes used in 1st version of the N501Y assay for large-scale testing for key mutations					
N501Y	SARS-CoV-2 501Y_mutant LNA probe	63.2	FAM-CCCAC+T+**T**+ATGG+TGTTGGT-BHQ1	0.05	1
	SARS-CoV2 N501 WT LNA probe	62.6	Quasar 670-CCCAC+T+**A**+ATGG+TGTTGGT-BHQ2	0.05	1

Probes used exclusively for multiplex RT-qPCR for small-scale testing for key mutations					
ΔH69/V70	SARS-CoV-2_ΔH69/V70 Zen probe	61.2	HEX-TTCCATGCT-Zen-ATCTCTGGGACCA-IABkFQ	0.05	5
E484K	SARS-CoV-2_484K_mutant MGB Cy5 probe	64.6	Cy5-TGGTGTT**A**AAGGTTTTAAT-MGB	0.15	5, 6

a A “+” before a nucleotide indicates the position of the locked nucleic acid (LNA)-modified base. Master mix ID indicates which primers and probes were used in the same master mix (see also the master mix setup described in the text). SNP mutations are marked in boldface type. MGB, minor groove binder; BHQ+, BHQplus modified probe. The 31 in E_Sarbeco31_F is an internal designation for the primer.

b While it is more common to use a BHQ1 quencher together with HEX, this system works well with a BHQ2 quencher.

c The volumes of oligonucleotides added to the master mix are valid for both the 96- and 384-well formats.

### Master mix setup.

The primers and probes were combined in different master mixes.

For master mixes 1 to 4 (Table 3), the mutations were detected using both the mutant probe and the wild-type probe for allelic discrimination analysis.

For master mixes 5 and 6 (Table 3), only probes targeting the mutations were used, and therefore, no allelic discrimination analysis was needed.

**(i) PCR conditions for small-scale testing in a 96-well format.** All PCR assays were developed on a Bio-Rad CFX 96 real-time PCR system. Master mixes 1 to 4 contained 12.5 μL of Luna universal probe one-step RT-qPCR kit reaction buffer (New England BioLabs Inc. [NEB]), 1.25 μL of Luna WarmStart RT enzyme mix, primers and probes (100 μM) (see Table 3 for the volumes), DNase/RNase-free water, and 5 μL of the template, for a total volume of 25 μL. Cycling conditions were as follows: reverse transcription at 55°C for 10 min and an initial denaturation step at 95°C for 3 min, followed by 45 cycles of denaturation and annealing/extension at 95°C for 15 s and 58°C for 30 s, respectively.

Master mixes 5 and 6 (multiplex RT-qPCR v.1 and v.2) contained 5 μL of 4× Luna probe one-step RT-qPCR mix with UDG (NEB), primers and probes (100 μM) (see Table 3 for the volumes), DNase/RNase-free water, and 5 μL of the template, for a total volume of 25 μL. Cycling conditions were as follows: an initial step at 25°C for 30 s, reverse transcription at 55°C for 10 min, and an initial denaturation step at 95°C for 1 min, followed by 45 cycles of denaturation and annealing/extension at 95°C for 10 s and 58°C for 60 s, respectively.

**(ii) Data analysis for the multiplexed RT-qPCRs used for small-scale testing.** Multiplexed RT-qPCR v.1 and v.2 contained probes targeting the mutations ΔH69/V70, 501Y, and 484K for master mix 5 and 501Y, 484K, and 452R for master mix 6. Cutoff values were used for the multiplexed RT-qPCRs to ensure the detection of only the mutation and not the WT sequence, as no WT probe was included in the mix. A sample was considered positive if it met the following criteria: a *C_T_* value of <38 and an RFU (relative fluorescence unit) value of >500 at a *C_T_* value of 45.

**(iii) PCR conditions for large-scale testing in a 384-well format.** For large-scale testing, the RT-qPCRs were run on a Bio-Rad CFX 384 real-time PCR system. The master mix contained 7.5 μL of Luna universal probe one-step RT-qPCR kit reaction buffer (New England BioLabs Inc.), 0.75 μL of Luna WarmStart RT enzyme mix, primers and probes (100 μM) (see Table 3 for the volumes), DNase/RNase-free water, and 5 μL of the template, for a total volume of 15 μL. Cycling conditions were the same as the ones described above for the 96-well format. Each patient sample was analyzed in four parallel reactions, using master mix 1 or 2 for the detection of ΔH69/V70 and N501Y, master mix 3 for the detection of E484K, and master mix 4 for the detection of L452R, and in the final well, the E-Sarbeco assay was used for the detection of wild-type SARS-CoV-2 (E gene) (Table 1). The four master mixes were arranged in a quadratic pattern to allow easy transfer from a 96-well plate to a 384-well plate. Probes included in master mixes 5 and 6 were not tested in the 384-well format.

**(iv) Data analysis using allelic discrimination analysis for large-scale testing.** PCR curves were evaluated using Bio-Rad CFX software, and *C_T_* and end RFU values were exported in CSV files. Files were imported into a laboratory database where all data analysis was performed. For ΔH69/V70, detection was based on *C_T_* values (*C_T_* cutoff values of between 12 and 38). For the N501Y, E484K, and L452R mutations, detection was based on allelic discrimination where the end RFU values were utilized to determine the presence of a mutation (see Table S1 in the supplemental material). A sample was considered positive if it met the following criteria: a *C_T_* value of <38 and an RFU value of >200 at a *C_T_* value of 45. The RFU cutoff value was used for the 384-well PCR format as a quality control step in case one of the probes in the allelic discrimination pair failed.

### Whole-genome sequencing.

Whole-genome sequences were generated by The Danish COVID-19 Genome Consortium (DCGC) from PCR-positive samples collected between 6 June and 11 July 2021. Samples were selected using *C_T_* cutoff values of between 30 and 38 (23). The bulk of the samples were sequenced using ARTIC Network tiled PCR scheme v3 via the COVIDseq assay (Illumina), ARTIC Network nCoV-2019 sequencing protocol v2 (https://doi.org/10.17504/protocols.io.bdp7i5rn) (Oxford Nanopore), or a custom DCGC protocol (https://doi.org/10.17504/protocols.io.bfc3jiyn) (Oxford Nanopore) adapted from the ARTIC Network protocol. Data preprocessing and consensus genome generation were performed using Illumina-specific (https://github.com/connor-lab/ncov2019-artic-nf) (v.1.3.0) or Oxford Nanopore-specific (https://github.com/artic-network/fieldbioinformatics) (v.1.2.1) consensus pipelines. Consensus genome mutation calling with reference to Wuhan-Hu-1/2019 (GenBank accession number MN908947) was performed with Nextclade CLI (https://github.com/nextstrain/nextclade) (v.1.2.0), and lineage designations were performed using Pangolin (https://github.com/cov-lineages/pangolin) (v.3.1.3) with the accompanying pangoLEARN model (https://github.com/cov-lineages/pangoLEARN) (v.1.2.6).

### RT-qPCR validation.

Nucleotide sequences corresponding to the S genes of consensus genomes derived from WGS were aligned using MAFFT version 7.480 (https://mafft.cbrc.jp), utilizing the fast Fourier transform (FFT) NS-2 algorithm with a maximum of 1,000 iterations (24, 25). Alignments were viewed and processed in Jalview 2.11.1.4 (https://www.jalview.org) (26), and codons encoding key mutations were extracted, translated, and compared to RT-qPCR results. From here, sensitivities, specificities, positive predictive values (PPVs), and negative predictive values (NPVs) were calculated for each set of primers and probes used in the RT-qPCR assays. Positive and negative predictive values were calculated according to the following formulas: PPV = (*Sen* × *Prev*)/[(*Sen* × *Prev*) + (1 − *Spec*) × (1 − *Prev*)] and NPV = [*Spec* × (1 − *Prev*)]/[*Spec* × (1 − *Prev*) + (1 − *Sen*) × *Prev*], where *Sen* is the sensitivity, *Spec* is the specificity, and *Prev* is the prevalence calculated from WGS consensus genomes. All analyses were performed in RStudio version 1.4.1717 using R version 4.1.1 and using the packages tidyverse (1.3.1), seqinr (4.2-8), lubridate (1.7.10), ggplot2 (3.3.4), cowplot (1.1.1), zoo (1.8-9), and ggpubr (0.4.0).

### Data analysis.

Standard curves were performed to determine the SARS-CoV-2 detection threshold for each assay and to calculate the viral load in each sample. The SARS-CoV-2 variant-specific TWIST controls with known concentrations (copies per microliter) were diluted 1:10 in a seven-step dilution series. The median *C_T_* values and the interquartile ranges were calculated based on biological duplicates with technical duplicates. The threshold was based on the intercept of the linear regression line of the TWIST control dilutions. Furthermore, the number of virus particles was estimated based on the logarithmic regression function of each assay’s TWIST control dilution series.

### Data availability.

WGS consensus genomes were uploaded to the publicly-accessible GISAID database (gisaid.org) and can be retrieved using the collection dates specified.

## Supplementary Material

Reviewer comments
