# Supplementary material for: Rapid and Flexible RT-qPCR Surveillance Platforms To Detect SARS-CoV-2 Mutations
Source: Microbiol Spectr. 2023 Jan 9;11(1):e03591-22. doi: 10.1128/spectrum.03591-22 (PMC9927487; doi:10.1128/spectrum.03591-22)
Supplement: Supplemental file 1 — Tables S1-S9. Download spectrum.03591-22-s0001.pdf, PDF file, 3.7 MB [file spectrum.03591-22-s0001.pdf]

Suppl. Table1 Data interpretation in detection of H69/70Δ, and detection of SNPs N501Y, E484K and L452R through allelic discrimination.

SARS-CoV-2 variant	H69/70Δ<br>HEX	501Y<br>Allele 1 -<br>Mutant<br>FAM	N501<br>Allele 2-<br>WT<br>Cy5	484K<br>Allele 1-<br>Mutant<br>FAM	E484<br>Allele 2 –<br>WT<br>Texas red	452R<br>Allele 1-<br>Mutant<br>HEX	L452<br>Allele 2 –<br>WT<br>Texas red
**WILDTYPE**	Ct ≥ 38	End RFU Allele2$_{(RFU>200)}$ > End RFU Allele1		End RFU Allele2$_{(RFU>200)}$ > End RFU Allele1		End RFU Allele2$_{(RFU>200)}$ > End RFU Allele1	
**Alpha**	**Ct < 38**	**End RFU Allele1$_{(RFU>200)}$ > End RFU Allele2**		End RFU Allele2$_{(RFU>200)}$ > End RFU Allele1		End RFU Allele2$_{(RFU>200)}$ > End RFU Allele1	
**Beta**	Ct ≥ 38	**End RFU Allele1$_{(RFU>200)}$ > End RFU Allele2**		**End RFU Allele1$_{(RFU>200)}$ > End RFU Allele2**		End RFU Allele2$_{(RFU>200)}$ > End RFU Allele1	
**Gamma**	Ct ≥ 38	**End RFU Allele1$_{(RFU>200)}$ > End RFU Allele2**		**End RFU Allele1$_{(RFU>200)}$ > End RFU Allele2**		End RFU Allele2$_{(RFU>200)}$ > End RFU Allele1	
**Delta**	Ct ≥ 38	End RFU Allele2$_{(RFU>200)}$ > End RFU Allele1		End RFU Allele2$_{(RFU>200)}$ > End RFU Allele1		**End RFU Allele1$_{(RFU>200)}$ > End RFU Allele2**	

RFU = Relative Fluorescence Units. Mutations are marked in bold.

Supplementary Table2 Sensitivity of the primer and probes targeting the four key mutations

PCR	Sample type[a]	Variant	H69/70 del	501Y	N501	484K	E484	452R	L452	del H69/V70[a] median CT (n=3)	IQR	copies/µl	501Y median CT (n=3)	IQR	copies/µl	N501 median CT (n=3)	IQR	copies/µl	484K			E484			452R		L452		
H69/70 & N501Y (501 MGB probes)	TWIST control 1x10^-3	Wt SARS-CoV2; Australian strain	no	no	yes					N/D	-	-	N/D	-	-	26.87	1.10	4761											
	TWIST control 1x10^-4	Wt SARS-CoV2; Australian strain	no	no	yes					N/D	-	-	N/D	-	-	30.10	0.93	520											
	TWIST control 1x10^-5	Wt SARS-CoV2; Australian strain	no	no	yes					N/D	-	-	N/D	-	-	33.59	0.45	48											
	TWIST control 1x10^-6	Wt SARS-CoV2; Australian strain	no	no	yes					N/D	-	-	N/D	-	-	N/D**	-	-											
	TWIST control 1x10^-7	Wt SARS-CoV2; Australian strain	no	no	yes					N/D	-	-	N/D	-	-	N/D	-	-											
	TWIST control 1x10^-8	Wt SARS-CoV2; Australian strain	no	no	yes					N/D	-	-	N/D	-	-	N/D	-	-											
	TWIST control 1x10^-3	SARS- CoV-2; B.1.1.7	yes	yes	no					27.21	0.68	5012	28.64	1.11	3898	N/D	-	-											
	TWIST control 1x10^-4	SARS- CoV-2; B.1.1.7	yes	yes	no					30.56	0.76	535	31.14	0.81	701	N/D	-	-											
	TWIST control 1x10^-5	SARS- CoV-2; B.1.1.7	yes	yes	no					34.32	0.64	44	34.86	0.63	54	N/D	-	-											
	TWIST control 1x10^-6	SARS- CoV-2; B.1.1.7	yes	yes	no					37.46	1.28	5	38.56	1.30	4	N/D	-	-											
	TWIST control 1x10^-7	SARS- CoV-2; B.1.1.7	yes	yes	no					N/D	-	-	N/D	-	-	N/D	-	-											
	TWIST control 1x10^-8	SARS- CoV-2; B.1.1.7	yes	yes	no					N/D	-	-	N/D	-	-	N/D	-	-											
	Patient sample A 1:10	SARS- CoV-2; B.1.1.7	yes	yes	no					22.68	0.52	-	23.57	1.65	-	N/D	-	-											
	Patient sample A 1:100	SARS- CoV-2; B.1.1.7	yes	yes	no					26.24	0.50	-	27.08	0.99	-	N/D	-	-											
	Patient sample A 1:1000	SARS- CoV-2; B.1.1.7	yes	yes	no					29.82	0.95	-	30.77	1.79	-	N/D	-	-											
	Patient sample B 1:10	SARS- CoV-2; B.1.1.7	yes	yes	no					23.19	1.10	-	24.04	1.13	-	N/D	-	-											
	Patient sample B 1:100	SARS- CoV-2; B.1.1.7	yes	yes	no					26.85	3.27	-	28.33	5.10	-	N/D	-	-											
	Patient sample B 1:1000	SARS- CoV-2; B.1.1.7	yes	yes	no					29.83	1.29	-	30.46	1.89	-	N/D	-	-											
	Patient sample	Neg. SARS-CoV-2								N/D			N/D																
	H2O	-								N/D			N/D																
**PCR**	**Sample type**	**Variant**	**H69/70 del**	**501Y**	**N501**	**484K**	**E484**	**452R**	**L452**	**H69/70 del**			**501Y**			**N501**			**484K** median CT (n=3)	**IQR**	**copies/µl**	**E484** median CT (n=3)	**IQR**	**copies/µl**	**452R**		**L452**		
E484K (MGB probes)	TWIST control 1x10^-3	Wt SARS-CoV2; Australian strain				no	yes												N/D	-	-	29.62	1.21	4768					
	TWIST control 1x10^-4	Wt SARS-CoV2; Australian strain				no	yes												N/D	-	-	32.635	0.59	524					
	TWIST control 1x10^-5	Wt SARS-CoV2; Australian strain				no	yes												N/D	-	-	35.775	0.23	52					
	TWIST control 1x10^-6	Wt SARS-CoV2; Australian strain				no	yes												N/D	-	-	39.05	0.85	5					
	TWIST control 1x10^-7	Wt SARS-CoV2; Australian strain				no	yes												N/D	-	-	N/D	-	-					
	TWIST control 1x10^-8	Wt SARS-CoV2; Australian strain				no	yes												N/D	-	-	N/D	-	-					
	TWIST control 1x10^-3	SARS- CoV-2; B.1.351				yes	no												28.88	0.91	5180	N/D	-	-					
	TWIST control 1x10^-4	SARS- CoV-2; B.1.351				yes	no												32.72	0.58	464	N/D	-	-					
	TWIST control 1x10^-5	SARS- CoV-2; B.1.351				yes	no												36.21	0.77	52	N/D	-	-					
	TWIST control 1x10^-6	SARS- CoV-2; B.1.351				yes	no												N/D**	-		N/D	-	-					
	TWIST control 1x10^-7	SARS- CoV-2; B.1.351				yes	no												N/D	-	-	N/D	-	-					
	TWIST control 1x10^-8	SARS- CoV-2; B.1.351				yes	no												N/D	-	-	N/D	-	-					
	TWIST control 1x10^-3	SARS- CoV-2; P.1				yes	no												29.03	0.29	5051	N/D	-	-					
	TWIST control 1x10^-4	SARS- CoV-2; P.1				yes	no												32.56	0.68	520	N/D	-	-					
	TWIST control 1x10^-5	SARS- CoV-2; P.1				yes	no												36.36	0.45	45	N/D	-	-					
	TWIST control 1x10^-6	SARS- CoV-2; P.1				yes	no												39.69	0.47	5	N/D	-	-					
	TWIST control 1x10^-7	SARS- CoV-2; P.1				yes	no												N/D	-	-	N/D	-	-					
	TWIST control 1x10^-8	SARS- CoV-2; P.1				yes	no												N/D	-	-	N/D	-	-					
	Patient sample	Neg. SARS-CoV-2																	N/D			N/D							
	H2O	-																	N/D			N/D							
**PCR**	**Sample type**	**Variant**	**H69/70 del**	**501Y**	**N501**	**484K**	**E484**	**452R**	**L452**	**H69/70 del**			**501Y**			**N501**			**484K**			**E484**			**452R** median CT (n=3)	**IQR**	**L452** median CT (n=3)	**IQR**	**copies/µl**
L452R (BHQplus probes)	patient sample 1x10^-1	SARS- CoV-2; B.1:617.2						no	yes																34.26	0.33	N/D	-	-
	patient sample 1x10^-2	SARS- CoV-2; B.1:617.2						no	yes																36.77	1.10	N/D	-	-
	patient sample 1x10^-3	SARS- CoV-2; B.1:617.2						no	yes																38.22	1.03	N/D	-	-
	patient sample 1x10^-4	SARS- CoV-2; B.1:617.2						no	yes																38.49	1.88	N/D	-	-
	patient sample 1x10^-5	SARS- CoV-2; B.1:617.2						no	yes																N/D	-	N/D	-	-
	TWIST control 1x10^-3	Wt SARS-CoV2; Australian strain																							N/D	-	28.26	0.29	4489
	TWIST control 1x10^-4	Wt SARS-CoV2; Australian strain																							N/D	-	31.21	0.49	586
	TWIST control 1x10^-5	Wt SARS-CoV2; Australian strain																							N/D	-	34.77	1.15	50
	TWIST control 1x10^-6	Wt SARS-CoV2; Australian strain																							N/D	-	38.17	2.59	5
	TWIST control 1x10^-7	Wt SARS-CoV2; Australian strain																							N/D	-	N/D[b]		
	Patient sample	Neg. SARS-CoV-2																							N/D		N/D		
	H2O	-																							N/D		N/D		

N/D = not detectable
[a] tested in duplicate
** CT value not detected in all six technical replicates. Set as detection threshold

Supplementary Tab.3 Specificity of primer and probes - large scale screening

PCR	Number	Sample type	Virus/Variant	H69/70 del	501Y	N501	484K	E484	452R	L452	H69/70 del			501Y			N501			484K			E484			452R			L452		
H69/70 & N501Y (501 LNA probes)	1	Patient sample	SARS-CoV2/B.1.298	yes	no	yes					29.12						30.01														
	2	Patient sample	SARS-CoV2/B.1.298	yes	no	yes					28.96						29.96														
	3	Patient sample	SARS-CoV-2/ B.1.1.7	yes	yes	no					27.00			28.15			N/D														
	4	Patient sample	SARS-CoV-2/ B.1.1.7	yes	yes	no					26.85			28.16			N/D														
	5	Patient sample	Human Coronavirus 229E								N/D			N/D			N/D														
	6	Patient sample	Human Coronavirus 229E								N/D			N/D			N/D														
	7	Patient sample	Human Coronavirus HKU1								N/D			N/D			N/D														
	8	Patient sample	Human Coronavirus HKU1								N/D			N/D			N/D														
	9	Patient sample	Human Coronavirus NL63								N/D			N/D			N/D														
	10	Patient sample	Human Coronavirus NL63								N/D			N/D			N/D														
	11	Patient sample	Human Coronavirus OC43								N/D			N/D			N/D														
	12	Patient sample	Human Coronavirus OC43								N/D			N/D			N/D														
	13	Virus Culture	Influenza A								N/D			N/D			N/D														
	14	Virus Culture	Influenza A								N/D			N/D			N/D														
	15	Virus Culture	Influenza B								N/D			N/D			N/D														
	16	Virus Culture	Influenza B								N/D			N/D			N/D														
	17	Patient sample	Adenovirus								N/D			N/D			N/D														
	18	Patient sample	Adenovirus								N/D			N/D			N/D														
	19	Patient sample	Rhinovirus								N/D			N/D			N/D														
	20	Patient sample	Rhinovirus								N/D			N/D			N/D														
	37	Patient sample	Neg. SARS-CoV-2								N/D			N/D			N/D														
	38	H2O	-								N/D			N/D			N/D														

PCR	Number	Sample type	Virus/Variant	H69/70 del	501Y	N501	484K	E484	452R	L452	H69/70 del (exp.1)	H69/70 del (exp.2)	H69/70 del (exp.3)	501Y (exp.1)	501Y (exp.2)	501Y (exp.3)	N501 (exp.1)	N501 (exp.2)	N501 (exp.3)	484K			E484			452R			L452		
H69/70 & N501Y (501 MGB probes)[a]	1	Patient sample	SARS-CoV2/B.1.298	yes	no	yes					31.72	30.05	29.69	N/D	N/D	N/D	31.10	30.14	29.82												
	2	Patient sample	SARS-CoV2/B.1.298	yes	no	yes					31.54	29.60	29.39	N/D	N/D	N/D	31.38	29.64	29.57												
	3	Patient sample	SARS-CoV-2/ B.1.1.7	yes	yes	no					27.53	26.75	26.55	27.31	27.94	27.3	N/D	N/D	N/D												
	4	Patient sample	SARS-CoV-2/ B.1.1.7	yes	yes	no					27.47	26.87	26.48	27.23	28.01	27.36	N/D	N/D	N/D												
	5	Patient sample	Human Coronavirus 229E								N/D	N/D	N/D	N/D	N/D	N/D	N/D	N/D	N/D												
	6	Patient sample	Human Coronavirus 229E								N/D	N/D	N/D	N/D	N/D	N/D	N/D	N/D	N/D												
	7	Patient sample	Human Coronavirus HKU1								N/D	N/D	N/D	N/D	N/D	N/D	N/D	N/D	N/D												
	8	Patient sample	Human Coronavirus HKU1								N/D	N/D	N/D	N/D	N/D	N/D	N/D	N/D	N/D												
	9	Patient sample	Human Coronavirus NL63								N/D	N/D	N/D	N/D	N/D	N/D	N/D	N/D	N/D												
	10	Patient sample	Human Coronavirus NL63								N/D	N/D	N/D	N/D	N/D	N/D	N/D	N/D	N/D												
	11	Patient sample	Human Coronavirus OC43								N/D	N/D	N/D	N/D	N/D	N/D	N/D	N/D	N/D												
	12	Patient sample	Human Coronavirus OC43								N/D	N/D	N/D	N/D	N/D	N/D	N/D	N/D	N/D												
	13	Virus Culture	Influenza A								N/D	N/D	N/D	N/D	N/D	N/D	N/D	N/D	N/D												
	14	Virus Culture	Influenza A								N/D	N/D	N/D	N/D	N/D	N/D	N/D	N/D	N/D												
	15	Virus Culture	Influenza B								N/D	N/D	N/D	N/D	N/D	N/D	N/D	N/D	N/D												
	16	Virus Culture	Influenza B								N/D	N/D	N/D	N/D	N/D	N/D	N/D	N/D	N/D												
	17	Patient sample	Adenovirus								N/D	N/D	N/D	N/D	N/D	N/D	N/D	N/D	N/D												
	18	Patient sample	Adenovirus								N/D	N/D	N/D	N/D	N/D	N/D	N/D	N/D	N/D												
	19	Patient sample	Rhinovirus								N/D	N/D	N/D	N/D	N/D	N/D	N/D	N/D	N/D												
	20	Patient sample	Rhinovirus								N/D	N/D	N/D	N/D	N/D	N/D	N/D	N/D	N/D												
	37	Patient sample	Neg. SARS-CoV-2								N/D	N/D	N/D	N/D	N/D	N/D	N/D	N/D	N/D												
	38	H2O	-								N/D	N/D	N/D	N/D	N/D	N/D	N/D	N/D	N/D												

PCR	Number	Sample type	Virus/Variant	H69/70 del	501Y	N501	484K	E484	452R	L452	H69/70 del			501Y			N501			484K (exp.1)	484K (exp.2)	484K (exp.3)	E484 (exp.1)	E484 (exp.2)	E484 (exp.3)	452R			L452		
E484K (MGB probes)[a]	1	Patient sample	SARS-CoV2/B.1.298				no	yes												N/D	N/D	N/D	31.37	30.5	31.41						
	2	Patient sample	SARS-CoV2/B.1.298				no	yes												N/D	N/D	N/D	31.96	30.55	31.32						
	3	Patient sample	SARS-CoV-2/ B1.1.7				no	yes												N/D	N/D	N/D	28.96	27.7	29.76						
	4	Patient sample	SARS-CoV-2/ B1.1.7				no	yes												N/D	N/D	N/D	28.60	27.62	28.78						
	5	Patient sample	SARS-CoV2/B.1.351																			27.49			N/D						
	6	Patient sample	SARS-CoV2/B.1.351																			28.2			N/D						
	7	Patient sample	Human Coronavirus 229E																	N/D	N/D	N/D	N/D	N/D	N/D						
	8	Patient sample	Human Coronavirus 229E																	N/D	N/D	N/D	N/D	N/D	N/D						
	9	Patient sample	Human Coronavirus HKU1																	N/D	N/D	N/D	N/D	N/D	N/D						
	10	Patient sample	Human Coronavirus HKU1																	N/D	N/D	N/D	N/D	N/D	N/D						
	11	Patient sample	Human Coronavirus NL63																	N/D	N/D	N/D	N/D	N/D	N/D						
	12	Patient sample	Human Coronavirus NL63																	N/D	N/D	N/D	N/D	N/D	N/D						
	13	Patient sample	Human Coronavirus OC43																	N/D	N/D	N/D	N/D	N/D	N/D						
	14	Patient sample	Human Coronavirus OC43																	N/D	N/D	N/D	N/D	N/D	N/D						
	15	Virus Culture	Influenza A																	N/D	N/D	N/D	N/D	N/D	N/D						
	16	Virus Culture	Influenza A																	N/D	N/D	N/D	N/D	N/D	N/D						
	17	Virus Culture	Influenza B																	N/D	N/D	N/D	N/D	N/D	N/D						
	18	Virus Culture	Influenza B																	N/D	N/D	N/D	N/D	N/D	N/D						
	19	Patient sample	Adenovirus																	N/D	N/D	N/D	N/D	N/D	N/D						
	20	Patient sample	Adenovirus																	N/D	N/D	N/D	N/D	N/D	N/D						
	21	Patient sample	Rhinovirus																	N/D	N/D	N/D	N/D	N/D	N/D						
	22	Patient sample	Rhinovirus																	N/D	N/D	N/D	N/D	N/D	N/D						
	22	Patient sample	Neg. SARS-CoV-2																	N/D	N/D	N/D	N/D	N/D	N/D						
	23	H2O	-																	N/D	N/D	N/D	N/D	N/D	N/D						

PCR	Number	Sample type	Virus/Variant	H69/70 del	501Y	N501	484K	E484	452R	L452	H69/70 del			501Y			N501			484K			E484			452R (exp.1)	452R (exp.2)	452R (exp.3)	L452 (exp.1)	L452 (exp.2)	L452 (exp.3)
L452R (BHQplus probes)[a]	1	Patient sample	SARS-CoV2 WT						no	yes																N/D	N/D	N/D	24.84	25.54	27.36
	2	Patient sample	SARS-CoV2/B.1.617.2						no	yes																26.31	26.36	24.84	N/D	N/D	N/D
	6	Patient sample	Human Coronavirus 229E																							N/D	N/D	N/D	N/D	N/D	N/D
	8	Patient sample	Human Coronavirus HKU1																							N/D	N/D	N/D	N/D	N/D	N/D
	10	Patient sample	Human Coronavirus NL63																							N/D	N/D	N/D	N/D	N/D	N/D
	12	Patient sample	Human Coronavirus OC43																							N/D	N/D	N/D	N/D	N/D	N/D
	14	Virus Culture	Influenza A																							N/D	N/D	N/D	N/D	N/D	N/D
	16	Virus Culture	Influenza B																							N/D	N/D	N/D	N/D	N/D	N/D
	19	Patient sample	Adenovirus																							N/D	N/D	N/D	N/D	N/D	N/D
	21	Patient sample	Rhinovirus																							N/D	N/D	N/D	N/D	N/D	N/D
	23	Patient sample	Neg. SARS-CoV-2																							N/D	N/D	N/D	N/D	N/D	N/D
	24	H2O	-																							N/D	N/D	N/D	N/D	N/D	N/D

[a] tested in three individual biological replicates

N/D = not detectable

Supplementary Tab. 4 Testing of samples with known genome sequence information in the multiplexed RT-qPCRs

PCR	Number	Sample type	Variant	delH69/V70	delH69/V70 (HEX/CT) exp.1	delH69/V70 (HEX/CT) exp. 2	delH69/V70 (HEX/CT) exp. 3	Median (n=3)	IQR	484K	484K (Cy5/CT) exp.1	484K (HEX/CT) exp. 2	484K (HEX/CT) exp. 3	Median (n=3)	IQR	501Y	501Y (FAM/CT) exp.1	501Y (FAM/CT) exp.2	501Y (FAM/CT) exp.3	Median (n=3)	IQR	452R	452R (Cy5/CT) exp.1	452R (HEX/CT) exp. 2	452R (HEX/CT) exp. 3	Median (n=3)	IQR
**Multiplexed RT-qPCR v.1**	1	Patient sample	B.1.525	yes	31.64	30.88	31.33	**31.33**	**0.38**	yes	31.09	31.95	32.21	**31.95**	**0.56**	no	N/D	N/D	N/D	-	-	no					
	2	Patient sample	B.1.525	yes	33.69	32.93	33.22	**33.22**	**0.38**	yes	33.17	34.02	34.27	**34.02**	**0.55**	no	N/D	N/D	N/D	-	-	no					
	3	Patient sample	B.1.525	yes	32.78	32.13	32.25	**32.25**	**0.33**	yes	32.42	33.62	33.26	**33.26**	**0.60**	no	N/D	N/D	N/D	-	-	no					
	4	Patient sample	B.1.525	yes	34.60	34.47	34.91	**34.60**	**0.22**	yes	34.03	35.68	35.74	**35.68**	**0.85**	no	N/D	N/D	N/D	-	-	no					
	5	Patient sample	B.1.525	yes	31.83	31.42	31.5	**31.50**	**0.20**	yes	31.41	32.82	32.54	**32.54**	**0.70**	no	N/D	N/D	N/D	-	-	no					
	6	Patient sample	B.1.1.7	yes	27.54	27.04	27.5	**27.50**	**0.25**	no	N/D	N/D	N/D	-	-	yes	28.94	29.39	28.69	**28.94**	**0.35**	no					
	7	Patient sample	B.1.1.7	yes	27.32	27.03	27.51	**27.32**	**0.24**	no	N/D	N/D	N/D	-	-	yes	28.15	28.91	28.25	**28.25**	**0.38**	no					
	8	Patient sample	B.1.1.7	yes	28.77	28.41	29.41	**28.77**	**0.50**	no	N/D	N/D	N/D	-	-	yes	30.12	31.36	30.31	**30.31**	**0.62**	no					
	9	Patient sample	B.1.1.7	yes	29.16	29.00	29.29	**29.16**	**0.15**	no	N/D	N/D	N/D	-	-	yes	29.95	31.05	30.13	**30.13**	**0.55**	no					
	10	Patient sample	B.1.1.7	yes	29.15	29.04	29.25	**29.15**	**0.11**	no	N/D	N/D	N/D	-	-	yes	29.96	31.06	29.66	**29.96**	**0.70**	no					
	11	Patient sample	B.1.351	no	N/D	N/D	N/D	-	-	yes	37.65	38.71	38.41	**38.41**	**0.53**	yes	37.65	38.43	37.42	**37.65**	**0.51**	no					
	14	Patient sample	B.1.351	no	42.42	N/D	N/D	-	-	yes	31.95	33.59	33.32	**33.32**	**0.82**	yes	31.8	32.98	32.25	**32.25**	**0.59**	no					
	15	Patient sample	B.1.351	no	43.69	N/D	N/D	-	-	yes	31.99	33.57	33.49	**33.49**	**0.79**	yes	31.77	33.07	32.12	**32.12**	**0.65**	no					
	16	Patient sample	B.1.351	no	41.76	N/D	N/D	-	-	yes	29.22	30.75	30.7	**30.70**	**0.77**	yes	29.31	30.14	29.4	**29.4**	**0.42**	no					
	17	Patient sample	B.1.351	no	N/D	N/D	N/D	-	-	yes	38.23	40.03	39.85	**39.85**	**0.90**	yes	38.28	40.18	38.96	**38.96**	**0.95**	no					
	18	Patient sample	P.2	no	N/D	N/D	N/D	-	-	yes	26.67	28.40	27.8	**27.80**	**0.87**	no	N/D	N/D	N/D	-	-	no					
	19	Patient sample	P.2	no	N/D	38.62	N/D	-	-	yes	27.5	29.47	28.85	**28.85**	**0.98**	no	41.24	N/D	44.79	-	-	no					
	20	Patient sample	P.2	no	N/D	N/D	N/D	-	-	yes	30.87	32.95	31.99	**31.99**	**1.04**	no	N/D	30.75	N/D	-	-	no					
	21	Patient sample	P.2	no	39.54	N/D	N/D	-	-	yes	28.57	31.50	30.4	**30.40**	**1.47**	no	N/D	N/D	N/D	-	-	no					
	22	Patient sample	P.2	no	42.97	N/D	N/D	-	-	yes	29.04	31.06	30.52	**30.52**	**1.01**	no	42.54	N/D	N/D	-	-	no					
	23	Patient sample	WT	no	N/D	N/D	N/D	-	-	no	N/D	N/D	N/D	-	-	no	N/D	N/D	N/D	-	-	no					
	19	Neg. control*			N/D	N/D	N/D				N/D	N/D	N/D				N/D	N/D	N/D								
	20	Water control			N/D	N/D	N/D				N/D	N/D	N/D				N/D	N/D	N/D								
**Multiplexed RT-qPCR v.2**	2	Patient sample	B.1.351	no						yes	33.45					yes	33.23					no	N/D				
	3	Patient sample	B.1.351	no						yes	26.63					yes	26.24					no	N/D				
	4	Patient sample	B.1.351	no						yes	34.15					yes	34.03					no	N/D				
	5	Patient sample	B.1.351	no						yes	29.01					yes	28.65					no	N/D				
	6	Patient sample	B.1.351	no						yes	30.03					yes	29.59					no	N/D				
	7	Patient sample	B.1.351	no						yes	33.20					yes	32.87					no	N/D				
	8	Patient sample	B.1.351	no						yes	29.91					yes	29.86					no	N/D				
	9	Patient sample	P.2	no						yes	29.20					no	N/D					no	N/D				
	10	Patient sample	P.2	no						yes	27.58					no	40.60					no	N/D				
	11	Patient sample	P.2	no						yes	28.53					no	N/D					no	N/D				
	12	Patient sample	P.2	no						yes	31.20					no	N/D					no	N/D				
	13	Patient sample	P.2	no						yes	30.26					no	44.96					no	N/D				
	14	Patient sample	P.2	no						yes	28.13					no	N/D					no	N/D				
	15	Patient sample	P.2	no						yes	32.11					no	N/D					no	N/D				
	16	Patient sample	P.2	no						yes	28.59					no	N/D					no	N/D				
	17	Patient sample	B.1.1.7	yes						no	N/D					yes	31.39					no	N/D				
	18	Patient sample	B.1.1.7	yes						no	N/D					yes	30.63					no	N/D				
	19	Patient sample	B.1.1.7	yes						no	N/D					yes	36.95					no	N/D				
	20	Patient sample	B.1.1.7	yes						no	N/D					yes	30.01					no	N/D				
	21	Patient sample	B.1.1.7	yes						no	N/D					yes	30.26					no	N/D				
	22	Patient sample	B.1.1.7	yes						no	N/D					yes	29.59					no	N/D				
	23	Patient sample	B.1.1.7	yes						no	N/D					yes	29.50					no	N/D				
	24	Patient sample	B.1.1.7	yes						no	N/D					yes	32.99					no	N/D				
	25	Patient sample	B.1.617	no						no**	N/D					no	N/D					yes	25.57				
	26	Patient sample	B.1.617	no						no**	37.30					no	N/D					yes	30.73				
	27	Patient sample	B.1.617	no						no**	N/D					no	N/D					yes	27.12				
	28	Patient sample	B.1.617	no						no**	34.75					no	N/D					yes	28.17				
	29	Patient sample	B.1.617	no						no**	35.47					no	N/D					yes	29.32				
	30	Patient sample	B.1.617	no						no**	43.18					no	N/D					yes	36.62				
	31	Patient sample	WT	no						no**	N/D					no	N/D					no	N/D				
	32	Neg. control*									N/D	N/D	N/D				N/D	N/A	N/D				N/D	N/D	N/D		
	33	Water control									N/D	N/D	N/D				N/D	N/D	N/D				N/D	N/D	N/D		

* negative SARS-CoV-2 sample
N/D = not detectable

Supplementary Tab.5 Specificity of the multiplexed RT-qPCR v.1 (small scale screening)

PCR	Number	Sample type	Virus/Variant	H69/V70	501Y	484K	452R	H69/V70 (HEX/CT) exp.1	H69/V70 (HEX/CT) exp.2	H69/V70 (HEX/CT) exp.3	501Y (FAM/CT) exp.1	501Y (FAM/CT) exp.2	501Y (FAM/CT) exp.3	484K (Cy5/CT) exp.1	484K (Cy5/CT) exp.2	484K (Cy5/CT) exp.3	452R
	3	Patient sample	SARS-CoV-2/ B.1.1.7	yes	yes	no		27.1	28.36	30.24	28.21	28.02	30.01	N/D	N/D	N/D	
	4	Patient sample	SARS-CoV-2/ B.1.1.7	yes	yes	no		27.05	28.36	30.16	28.01	27.91	29.93	N/D	N/D	N/D	
	5	Patient sample	Human Coronavirus 229E					N/D	N/D	N/D	N/D	N/D	N/D	N/D	N/D	N/D	
	6	Patient sample	Human Coronavirus 229E					N/D	N/D	N/D	N/D	N/D	N/D	N/D	N/D	N/D	
	7	Patient sample	Human Coronavirus HKU1					N/D	N/D	N/D	N/D	N/D	N/D	N/D	N/D	N/D	
	8	Patient sample	Human Coronavirus HKU1					38.51	N/D	N/D	N/D	N/D	N/D	N/D	N/D	N/D	
	9	Patient sample	Human Coronavirus NL63					N/D	N/D	N/D	N/D	N/D	N/D	N/D	N/D	N/D	
	10	Patient sample	Human Coronavirus NL63					N/D	N/D	N/D	N/D	N/D	N/D	N/D	N/D	N/D	
	11	Patient sample	Human Coronavirus OC43					N/D	N/D	N/D	N/D	N/D	N/D	N/D	N/D	39.82	
	12	Patient sample	Human Coronavirus OC43					N/D	38.64	N/D	N/D	N/D	N/D	N/D	N/D	42.83	
	13	Virus Culture	Influenza A					N/D	N/D	N/D	N/D	N/D	N/D	N/D	N/D	N/D	
	14	Virus Culture	Influenza A					N/D	N/D	N/D	N/D	N/D	N/D	N/D	N/D	N/D	
	15	Virus Culture	Influenza B					N/D	N/D	N/D	N/D	N/D	N/D	N/D	N/D	N/D	
	16	Virus Culture	Influenza B					N/D	39.69	N/D	N/D	N/D	N/D	N/D	N/D	N/D	
	17	Patient sample	Adenovirus					N/D	N/D	N/D	N/D	N/D	N/D	N/D	N/D	N/D	
	18	Patient sample	Adenovirus					N/D	N/D	N/D	N/D	N/D	N/D	N/D	N/D	N/D	
	19	Patient sample	Rhinovirus					N/D	N/D	N/D	N/D	N/D	N/D	N/D	N/D	N/D	
	20	Patient sample	Rhinovirus					N/D	N/D	N/D	N/D	N/D	N/D	N/D	N/D	N/D	
	37	Patient sample	Neg. SARS-CoV-2					N/D	N/D	N/D	N/D	N/D	N/D	N/D	N/D	N/D	
	38	H2O	-					N/D	N/D	N/D	N/D	N/D	N/D	N/D	N/D	N/D	

N/D = not detectable

Supplementary Tab. 6 Sensitivity of the primer and probes targeting the four key mutations - small scale screening

PCR	Sample type[a]	Variant	H69/70 del	501Y	484K	del H69/V70[a] median CT (n=3)	IQR	copies/µl	501Y median CT (n=3)	IQR	copies/µl	484K median CT (n=3)	IQR	copies/µl
multiplexed RT-qPCR v.1	TWIST control 1x10^-3	SARS-CoV2; Australian strain, WT	no	no	no	N/D	-	-	N/D	-	-	N/D	-	-
	TWIST control 1x10^-4	SARS-CoV2; Australian strain, WT	no	no	no	N/D	-	-	N/D	-	-	N/D	-	-
	TWIST control 1x10^-5	SARS-CoV2; Australian strain, WT	no	no	no	N/D	-	-	N/D	-	-	N/D	-	-
	TWIST control 1x10^-6	SARS-CoV2; Australian strain, WT	no	no	no	N/D	-	-	N/D	-	-	N/D	-	-
	TWIST control 1x10^-7	SARS-CoV2; Australian strain, WT	no	no	no	N/D	-	-	N/D	-	-	N/D	-	-
	TWIST control 1x10^-8	SARS-CoV2; Australian strain, WT	no	no	no	N/D	-	-	N/D	-	-	N/D	-	-
	TWIST control 1x10^-3	SARS- CoV-2; B.1.1.7	yes	yes	no	30.45	0.26	4810	31.25	0.30	4663	N/D	-	-
	TWIST control 1x10^-4	SARS- CoV-2; B.1.1.7	yes	yes	no	34.22	0.06	542	35.04	0.08	573	N/D	-	-
	TWIST control 1x10^-5	SARS- CoV-2; B.1.1.7	yes	yes	no	38.39	0.88	48	39.57	1.46	47	N/D	-	-
	TWIST control 1x10^-6	SARS- CoV-2; B.1.1.7	yes	yes	no	N/D	-	-	N/D	-	-	N/D	-	-
	TWIST control 1x10^-7	SARS- CoV-2; B.1.1.7	yes	yes	no	N/D	-	-	N/D	-	-	N/D	-	-
	TWIST control 1x10^-8	SARS- CoV-2; B.1.1.7	yes	yes	no	N/D	-	-	N/D	-	-	N/D	-	-
	TWIST control 1x10^-3	SARS- CoV-2; B.1351	no	yes	yes	N/D	-	-	31.99	0.35	4635	31.33	0.12	4963
	TWIST control 1x10^-4	SARS- CoV-2; B.1351	no	yes	yes	N/D	-	-	35.04	0.47	582	34.60	0.17	509
	TWIST control 1x10^-5	SARS- CoV-2; B.1351	no	yes	yes	N/D	-	-	38.76	0.91	46	37.94	0.35	50
	TWIST control 1x10^-6	SARS- CoV-2; B.1351	no	yes	yes	N/D	-	-	N/D	-	-	N/D	-	-
	TWIST control 1x10^-7	SARS- CoV-2; B.1351	no	yes	yes	N/D	-	-	N/D	-	-	N/D	-	-
	TWIST control 1x10^-8	SARS- CoV-2; B.1351	no	yes	yes	N/D	-	-	N/D	-	-	N/D	-	-
	TWIST control 1x10^-3	SARS- CoV-2; P.1		yes	yes	N/D	-	-	30.39	0.34	5334.00	30.135	0.10	5161
	TWIST control 1x10^-4	SARS- CoV-2; P.1		yes	yes	N/D	-	-	34.09	0.10	442.00	33.78	0.09	471
	TWIST control 1x10^-5	SARS- CoV-2; P.1		yes	yes	N/D	-	-	37.23	0.26	53.00	37.15	0.03	52
	TWIST control 1x10^-6	SARS- CoV-2; P.1		yes	yes	N/D	-	-	N/D	-	-	N/D**	-	-
	TWIST control 1x10^-7	SARS- CoV-2; P.1		yes	yes	N/D	-	-	N/D	-	-	N/D	-	-
	TWIST control 1x10^-8	SARS- CoV-2; P.1		yes	yes	N/D	-	-	N/D	-	-	N/D	-	-
	Patient sample<br>H2O	Neg. SARS-CoV-2<br>-				N/D<br>N/D			N/D<br>N/D			N/D<br>N/D		

N/D = not detectable
** CT value not detected in all six technical replicates. Set as detection threshold

Supplementary Tab. 7 Testing of samples with known genome sequence information for the H69/V70 and N501Y mutation

						Mutliplex PCR										
**PCR**	**Number**	**Sample type**	**Variant**	**H69/V70 del**	**501Y**	**H69/V70 (HEX, CT) exp. 1**	**H69/V70 (HEX, CT) exp. 2**	**H69/V70 (HEX, CT) exp. 3**	**Median (n=3)**	**IQR**	**501Y (FAM, CT) exp. 1**	**501Y (FAM, CT) exp. 2**	**501Y (FAM, CT) exp. 3**	**Median (n=3)**	**IQR**	**Allelic discrimination analysis N510Y[c]**
**H69/70 & N501Y (501 LNA probes)[a]**	2	SARS-CoV-2 virus culture	B.1.298	yes	no	29.93										N501
	3	SARS-CoV-2 virus culture	B.1.298	yes	no	31.61										N501
	4	SARS-CoV-2 virus culture	B.1.298	yes	no	32.74										N501
	5	SARS-CoV-2 virus culture	B.1.298	yes	no	31.91										N501
	6	SARS-CoV-2 virus culture	B.1.298	yes	no	32										N501
	7	SARS-CoV-2 virus culture	B.1.298	yes	no	28.16										N501
	8	SARS-CoV-2 virus culture	B.1.298	yes	no	27.02										N501
	9	SARS-CoV-2 virus culture	B.1.298	yes	no	27.38										N501
	10	SARS-CoV-2 virus culture	B.1.258	yes	no	27.46										N501
	11	SARS-CoV-2 virus culture	Wt	no	no	N/D**										N501
	12	Patient sample	B.1.258	yes	no	31.68										N501
	13	Patient sample	B.1.258	yes	no	26.62										N501
	14	Patient sample	B.1.258	yes	no	30.92										N501
	15	Patient sample	B.1.258	yes	no	29.44										N501
	16	Patient sample	B.1.258	yes	no	30.79										N501
	17	Patient sample	B.1.298	yes	no	25.19										N501
	18	Patient sample	B.1.298	yes	no	27.92										N501
	19	Patient sample	B.1.298	yes	no	26.65										N501
	20	Patient sample	B.1.298	yes	no	27.16										N501
	21	Patient sample	B.1.298	yes	no	22.67										N501
	22	Patient sample	B.1.1.7	yes	yes	28.64										501Y
	23	Patient sample	B.1.1.7	yes	yes	24.59										501Y
	24	Patient sample	B.1.1.7	yes	yes	25.35										501Y
	25	Patient sample	B.1.1.7	yes	yes	24.29										501Y
	26	Patient sample	B.1.1.7	yes	yes	30.45										501Y
	27	Patient sample	B.1.1.7	yes	yes	24.04										501Y
	28	Patient sample	B.1.1.7	yes	yes	27.11										501Y
	29	Patient sample	B.1.1.7	yes	yes	27.34										501Y
	30	Patient sample	B.1.1.7	yes	yes	28.25										501Y
	31	Patient sample	B.1.1.7	yes	yes	25.51										501Y
	32	Patient sample	B.1.1.7	yes	yes	25.34										501Y
	33	Patient sample	B.1.1.7	yes	yes	27.62										501Y
	34	Patient sample	B.1.1.7	yes	yes	26.87										501Y
	35	Patient sample	B.1.1.7	yes	yes	27.66										501Y
	36	Patient sample	B.1.1.7	yes	yes	34.51										501Y
	37	Patient sample	B.1.1.7	yes	yes	28.69										501Y
	38	Patient sample	B.1.1.7	yes	yes	28.69										501Y
	39	Patient sample	B.1.1.7	yes	yes	28.69										501Y
**H69/70 & N501Y (501 MGB probes)[b]**	1	Patient sample	B.1.351	no	yes	N/D	N/D	N/D		N/D	30.32	31.63	31.9	**31.63**	**0.79**	501Y
	2	Patient sample	B.1.351	no	yes	N/D	N/D	N/D		N/D	23.52	24.28	24.98	**24.28**	**0.73**	501Y
	3	Patient sample	B.1.351	no	yes	N/D	N/D	N/D		N/D	28.7	32.27	32.32	**32.27**	**1.81**	501Y
	4	Patient sample	B.1.351	no	yes	N/D	N/D	N/D		N/D	25.52	26.58	27.14	**26.58**	**0.81**	501Y
	5	Patient sample	B.1.351	no	yes	N/D	N/D	N/D		N/D	29.7	30.47	30.97	**30.47**	**0.63**	501Y
	6	Patient sample	B.1.351	no	yes	N/D	N/D	N/D		N/D	27.05	28.28	28.5	**28.28**	**0.73**	501Y
	7	Patient sample	B.1.351	no	yes	N/D	N/D	N/D		N/D	25.37	27.81	28.18	**27.81**	**1.41**	501Y
	8	Patient sample	B.1.1.7	yes	yes	27.23	28.71	28.98	**28.71**	**0.88**	27.38	29.64	29.75	**29.64**	**1.19**	501Y
	9	Patient sample	B.1.1.7	yes	yes	31.57	27.68	28.3	**28.3**	**1.95**	32.5	28.54	29.06	**29.06**	**1.98**	501Y
	10	Patient sample	B.1.1.7	yes	yes	26.13	28.16	28.36	**28.16**	**1.12**	26.25	29.09	29.17	**29.09**	**1.46**	501Y
	11	Patient sample	B.1.1.7	yes	yes	27.1	28.35	28.69	**28.35**	**0.80**	27.36	29.05	29.33	**29.05**	**0.98**	501Y
	12	Patient sample	B.1.1.7	yes	yes	26.04	27.77	27.72	**27.72**	**0.86**	26.11	28.25	28.16	**28.16**	**1.07**	501Y
	13	Patient sample	B.1.1.7	yes	yes	25.86	28.09	27.98	**27.98**	**1.12**	26.04	28.69	28.28	**28.28**	**1.33**	501Y
	14	Patient sample	B.1.1.7	yes	yes	26.12	28.54	28.37	**28.37**	**1.21**	26.03	29.77	29.35	**29.35**	**1.87**	501Y
	15	Patient sample	B.1.1.7	yes	yes	25.23	27.45	27.23	**27.23**	**1.11**	25.14	28.36	28.06	**28.06**	**1.61**	501Y
	16	Patient sample	B.1.1.7	yes	yes	24.56	26.52	26.69	**26.52**	**1.07**	24.53	27.55	27.56	**27.55**	**1.52**	501Y
	17	Patient sample	B.1.1.7	yes	yes	26.26	27.25	27.77	**27.25**	**0.75**	26.38	28.1	28.44	**28.1**	**1.03**	501Y
	18	Neg. control*				N/D	N/D	N/D	N/D	N/D	N/D	N/D	N/D	N/D	N/D	N/D
	19	Water control				N/D	N/D	N/D	N/D	N/D	N/D	N/D	N/D	N/D	N/D	N/D

* Neg. control negative SARS-CoV-2 patient sample
N/D = not detectable
[a] tested in one biological replicate
[b] tested in three biological replicates
[c] for the H69/V70 & 501 MGB probes the results for the allelic discrimination analysis was the same for all three runs

Supplementary Tab. 8 Testing of samples with known sequence information for the L452R mutation

PCR	Number	Sample type	Variant	452R	452R (HEX/CT) exp.1	452R (HEX/CT) exp. 2	452R (HEX/CT) exp. 3	452R (HEX/CT) exp. 4	Median (n=3)	IQR	Allelic discrimination analysis[b]
**L452R (BHQplus probes)[a]**	1	Patient sample	B.1.617	yes	24.15	#	#	#			452R
	2	Patient sample	B.1.617	yes	30.06	#	#	#			452R
	3	Patient sample	B.1.617	yes	25.79	#	#	#			452R
	4	Patient sample	B.1.617	yes	26.64	#	#	#			452R
	5	Patient sample	B.1.617	yes	27.99	#	#	#			452R
	6	Patient sample	B.1.617	yes	#	25.99	26.31	27.36	**26.31**	**0.69**	452R
	7	Patient sample	B.1.617	yes	#	28.67	29.17	29.05	**29.05**	**0.25**	452R
	8	Patient sample	B.1.617	yes	#	35.43	35.77	36.65	**35.77**	**0.61**	452R
	9	Patient sample	B.1.617	yes	#	31.69	31.33	32.38	**31.69**	**0.53**	452R
	10	Patient sample	B.1.617	yes	#	33.74	34.58	34.64	**34.58**	**0.45**	452R
	11	Patient sample	B.1.617	yes	#	23.88	23.58	24.5	**23.88**	**0.46**	452R
	14	Patient sample	B.1.351	no	N/D	N/D	N/D				L452
	15	Patient sample	B.1.351	no	N/D	N/D	N/D				L452
	16	Patient sample	B.1.1.7	no	N/D	N/D	N/D				L452
	17	Patient sample	B.1.1.7	no	N/D	N/D	N/D				L452
	18	Patient sample	WT	no	N/D	N/D	N/D				L452
	19	Neg. control*			N/D	N/D	N/D	N/D			N/D
	20	Water control			N/D	N/D	N/D	N/D			N/D

* Neg. control negative SARS-CoV-2 patient sample

N/D = not detectable

" samples not included into this experiment

[a] tested in three biological replicates

[b]for the L452R BHQplus probes the results for the allelic discremination analysis was the same for all three runs

Supplementary Tab. 9 Testing of samples with known genome sequence information for the E484K mutation

PCR	Number	Sample type	Variant	484K	E484K (FAM/CT) exp. 1	E484K (FAM/CT) exp. 2	E484K (FAM/CT) exp. 3	Median (n=2-3)	IQR	Allelic discrimination analysis[b]
E484K (MGB probes)[a]	1	Patient sample	B.1.351	yes	33.28	31.43	32.15	**32.15**	**0.93**	484K
	2	Patient sample	B.1.351	yes	25.80	25.05	24.71	**25.05**	**0.54**	484K
	3	Patient sample	B.1.351	yes	28.61	27.18	#	**27.90**	**0.71**	484K
	4	Patient sample	B.1.351	yes	32.81	31.10	#	**31.96**	**0.85**	484K
	5	Patient sample	B.1.351	yes	29.90	28.23	#	**29.07**	**0.83**	484K
	6	Patient sample	B.1.351	yes	29.94	27.16	28.51	**28.51**	**1.39**	484K
	10	Patient sample	P2	yes	28.51	27.00	27.51	**27.51**	**0.75**	484K
	11	Patient sample	P2	yes	27.07	25.65	26.03	**26.03**	**0.71**	484K
	12	Patient sample	P2	yes	27.93	26.63	26.54	**26.63**	**0.70**	484K
	13	Patient sample	P2	yes	30.53	29.26	29.48	**29.48**	**0.64**	484K
	14	Patient sample	P2	yes	30.09	28.55	28.41	**28.55**	**0.84**	484K
	15	Patient sample	P2	yes	27.58	25.94	26.21	**26.21**	**0.82**	484K
	16	Patient sample	P2	yes	31.66	30.82	31.63	**31.63**	**0.42**	484K
	17	Patient sample	P2	yes	29.05	27.02	28.58	**28.58**	**1.02**	484K
	18	Patient sample	P2	yes	30.17	28.38	29.93	**29.93**	**0.90**	484K
	19	Patient sample	P2	yes	28.71	27.06	27.46	**27.46**	**0.83**	484K
	20	Patient sample	B.1.525	yes	23.54	22.00	22.61	**22.61**	**0.77**	484K
	21	Patient sample	B.1.525	yes	30.23	28.73	29.05	**29.05**	**0.75**	484K
	22	Patient sample	B.1.525	yes	23.59	22.08	22.45	**22.45**	**0.75**	484K
	23	Patient sample	B.1.525	yes	29.10	27.35	27.74	**27.74**	**0.88**	484K
	24	Patient sample	B.1.525	yes	29.10	27.89	29.7	**29.10**	**0.90**	484K
	25	Patient sample	B.1.525	yes	26.62	25.33	26.02	**26.02**	**0.65**	484K
	26	Patient sample	B.1.525	yes	26.70	25.03	26.06	**26.06**	**0.83**	484K
	27	Patient sample	B.1.525	yes	27.23	25.73	26.38	**26.38**	**0.75**	484K
	28	Patient sample	B.1.525	yes	25.46	23.43	24.62	**24.62**	**1.02**	484K
	29	Patient sample	B.1.525	yes	26.04	24.36	25.74	**25.74**	**0.84**	484K
	30	Patient sample	B.1.1.7	no	N/D	N/D	N/D	N/D	N/D	E484
	31	Patient sample	B.1.1.7	no	N/D	N/D	N/D	N/D	N/D	E484
	32	Neg. control*			N/D	N/D	N/D			N/D
	33	Water control			N/D	N/D	N/D			N/D
