# Supplementary material for: Rapid and Flexible RT-qPCR Surveillance Platforms To Detect SARS-CoV-2 Mutations
Source: Microbiol Spectr. 2023 Jan 9;11(1):e03591-22. doi: 10.1128/spectrum.03591-22 (PMC9927487; doi:10.1128/spectrum.03591-22)
Supplement: Supplemental file 2 — Fig. S1. Download spectrum.03591-22-s0002.pdf, PDF file, 0.4 MB [file spectrum.03591-22-s0002.pdf]

## Supplementary Fig.1

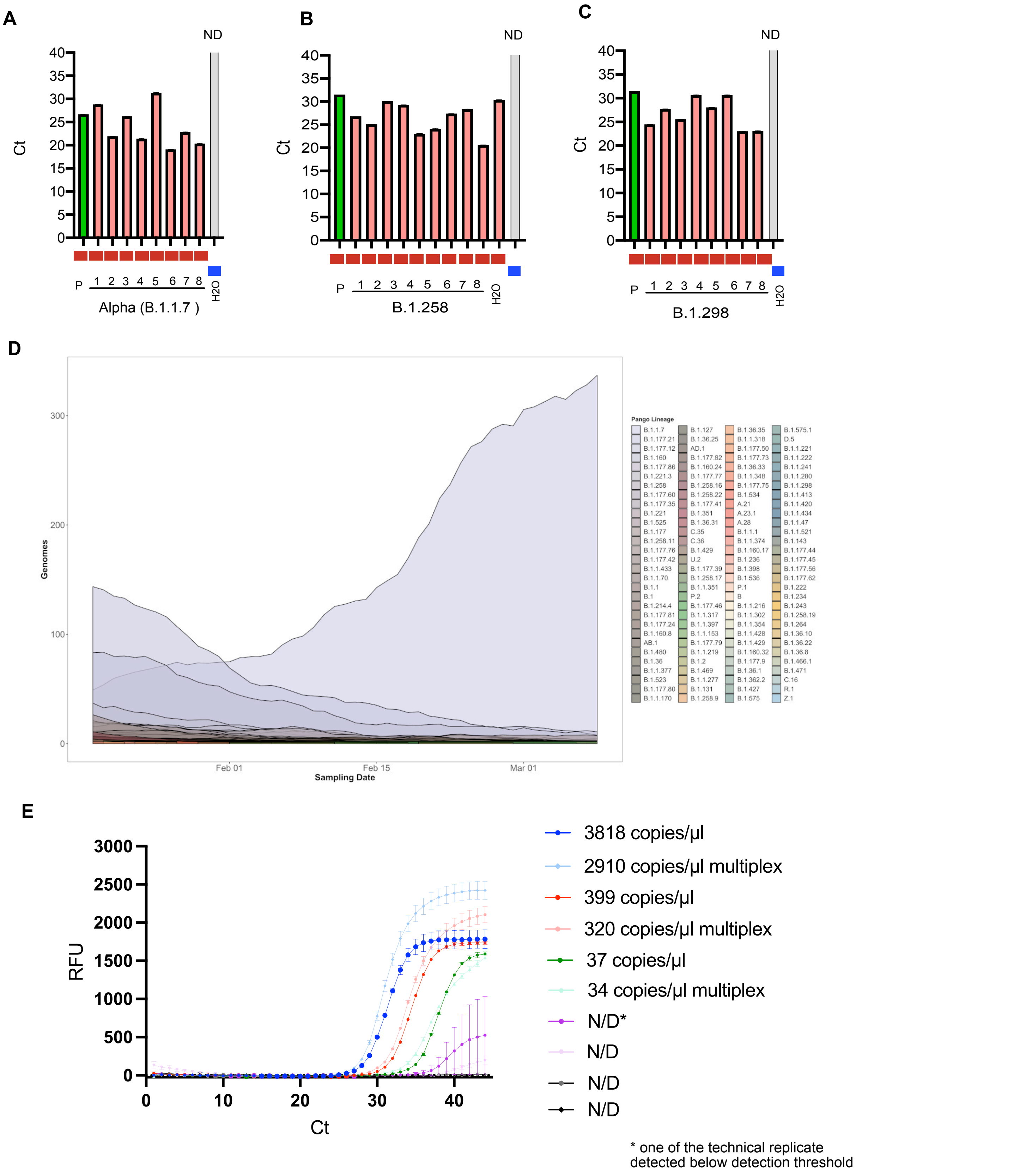


**Supplementary Figure 1 Detection of the H69/70V deletion by RT-qPCR. A-C)** Detection of the ΔH69/V70 in patient sample with known whole genome sequence information identified as Alpha (B.1.1.7), B.1.258 and B.1.298 variants (red bars). Positive control (green bar) of sample with known sequence information positive for the ΔH69/70 and the negative control (grey bar). **D)** Prevalence of all SARS-CoV-2 variants in Denmark (based on pangolin lineage assignments using WGS-derived consensus genomes) from 12$^{th}$ Jan 2021 to 8$^{th}$ Mar 2021. **E)** Dilution of the TWIST control (WT SARS-CoV-2) and detection of the H69/V70 WT sequence by the ΔH69/V70 RT-qPCR or the multiplexed ΔH69/V70_E-sarbeco RT-qPCR. Arrow bars in **E** indicate SEM for two technical replicates.
