# Supplementary material for: Rapid and Flexible RT-qPCR Surveillance Platforms To Detect SARS-CoV-2 Mutations
Source: Microbiol Spectr. 2023 Jan 9;11(1):e03591-22. doi: 10.1128/spectrum.03591-22 (PMC9927487; doi:10.1128/spectrum.03591-22)
Supplement: Supplemental file 3 — Fig. S2. Download spectrum.03591-22-s0003.pdf, PDF file, 0.2 MB [file spectrum.03591-22-s0003.pdf]

Supplementary Fig.2

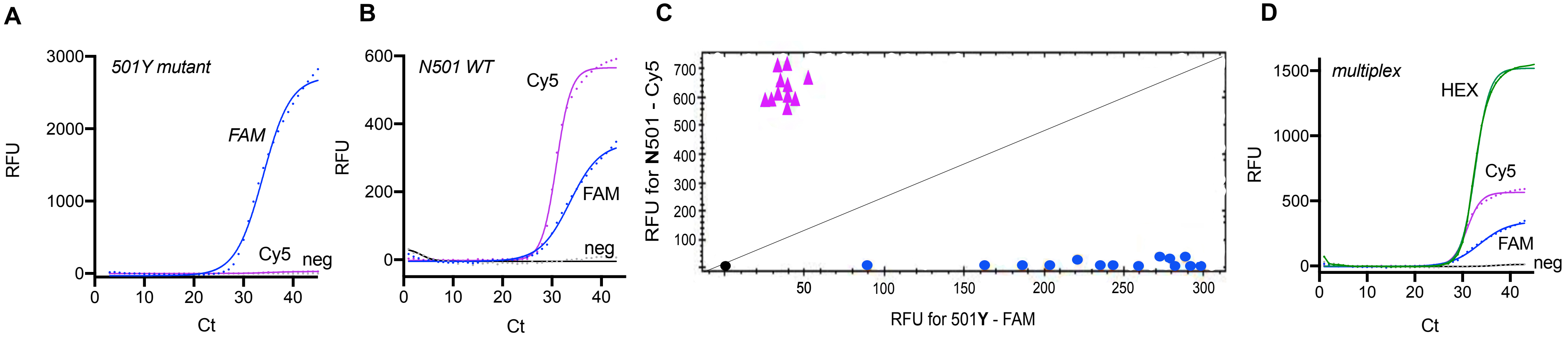

Supplementary Figure 2 LNA-modified probes detecting the N501Y mutation. A-B) LNA probes detecting the 501Y mutation and N501 WT sequence respectively. **C)** Allelic discrimination analysis to differentiate between the 510Y mutation and N501WT sequence. **D)** Multiplexed PCR to detect the ΔH69/V70 mutation and the N501Y mutation.
