# Supplementary material for: Rapid and Flexible RT-qPCR Surveillance Platforms To Detect SARS-CoV-2 Mutations
Source: Microbiol Spectr. 2023 Jan 9;11(1):e03591-22. doi: 10.1128/spectrum.03591-22 (PMC9927487; doi:10.1128/spectrum.03591-22)
Supplement: Reviewer comments [file reviewer-comments.pdf]

AMERICAN SOCIETY FOR MICROBIOLOGY | Microbiology Spectrum

Rapid and flexible RT-qPCR surveillance platforms to detect SARS-CoV-2 mutations

Katja Spiess, Vithiagaran Gunalan, Ellinor Marving, Sofie Nielsen, Michelle Jørgensen, Anna Fomsgaard, Line Nielsen, Alonzo Alfaro-Núñez, Søren Karst, The Danish COVID-19 Genome Consortium (DCGC), Shila Mortensen, Morten Rasmussen, Ria Lassaunière, Maiken Rosenstierne, Charlotta Polacek, Jannik Fonager, Arieh Cohen, Claus Nielsen, and Anders Fomsgaard

Corresponding Author(s): Katja Spiess, Statens Serum Institut

Review Timeline:

Submission Date:	September 7, 2022
Accepted:	November 21, 2022

Editor: Jonathan Jacobs

Reviewer(s): Disclosure of reviewer identity is with reference to reviewer comments included in decision letter(s). The following individuals involved in review of your submission have agreed to reveal their identity: Miguel Angel Garcia Bereguiain (Reviewer #2)

Transaction Report:

(Note: With the exception of the correction of typographical or spelling errors that could be a source of ambiguity, letters and reports are not edited. The original formatting of letters and referee reports may not be reflected in this compilation.)

DOI: https://doi.org/10.1128/spectrum.03591-22

November 21, 2022

Dr. Katja Spiess
Statens Serum Institut
Artillerivej 5
Copenhagen
Denmark

Re: Spectrum03591-22 (Rapid and flexible RT-qPCR surveillance platforms to detect SARS-CoV-2 mutations)

Dear Dr. Katja Spiess:

It's with great pleasure that I'm able to let you know that this manuscript has been accepted for publication in Microbiology Spectrum. The reviewers were impressed by the scale of the work and the impact it had on the Danish public health response during the emergence of the Omicron variant. My only request is that you add to the Discussion any thoughts you may have about expanding this assay to include other new variants. Otherwise, the paper is accepted as is.

Your manuscript has been accepted, and I am forwarding it to the ASM Journals Department for publication. You will be notified when your proofs are ready to be viewed.

The ASM Journals program strives for constant improvement in our submission and publication process. Please tell us how we can improve your experience by taking this quick Author Survey.

As an open-access publication, Spectrum receives no financial support from paid subscriptions and depends on authors' prompt payment of publication fees as soon as their articles are accepted. You will be contacted separately about payment when the proofs are issued; please follow the instructions in that e-mail. Arrangements for payment must be made before your article is published. For a complete list of **Publication Fees**, including supplemental material costs, please visit ourwebsite.

ASM policy requires that data be available to the public upon online posting of the article, so please verify all links to sequence records, if present, and make sure that each number retrieves the full record of the data. If a new accession number is not linked or a link is broken, provide production staff with the correct URL for the record. If the accession numbers for new data are not publicly accessible before the expected online posting of the article, publication of your article may be delayed; please contact the ASM production staff immediately with the expected release date.

Corresponding authors may join or renew ASM membership to obtain discounts on publication fees. Need to upgrade your membership level? Please contact Customer Service at Service@asmusa.org.

Thank you for submitting your paper to Spectrum.

Sincerely,

Jonathan Jacobs
Editor, Microbiology Spectrum

Journals Department
American Society for Microbiology
1752 N St., NW
Washington, DC 20036
E-mail: spectrum@asmusa.org

Supplemental Material: Accept
